# Author Correction: Highly efficient, heat dissipating, stretchable organic light-emitting diodes based on a MoO_3_/Au/MoO_3_ electrode with encapsulation

**DOI:** 10.1038/s41467-021-27720-8

**Published:** 2021-12-16

**Authors:** Dae Keun Choi, Dong Hyun Kim, Chang Min Lee, Hassan Hafeez, Subrata Sarker, Jun Su Yang, Hyung Ju Chae, Geon-Woo Jeong, Dong Hyun Choi, Tae Wook Kim, Seunghyup Yoo, Jinouk Song, Boo Soo Ma, Taek-Soo Kim, Chul Hoon Kim, Hyun Jae Lee, Jae Woo Lee, Donghyun Kim, Tae-Sung Bae, Seung Min Yu, Yong-Cheol Kang, Juyun Park, Kyoung-Ho Kim, Muhammad Sujak, Myungkwan Song, Chang-Su Kim, Seung Yoon Ryu

**Affiliations:** 1 Division of Display and Semiconductor Physics, Display Convergence, College of Science and Technology, Korea University Sejong Campus, Sejong City, Republic of Korea; 2 Department of Applied Physics, Korea University Sejong Campus, Sejong City, Republic of Korea; 3 E-ICT–Culture-Sports Convergence Track, Korea University Sejong Campus, Sejong City, Republic of Korea; 4 School of Electrical Engineering, Korea Advanced Institute of Science and Technology (KAIST), Daejeon, Republic of Korea; 5 Department of Mechanical Engineering, Korea Advanced Institute of Science and Technology (KAIST), Daejeon, Republic of Korea; 6 Department of Advanced Materials Chemistry, College of Science and Technology, Korea University Sejong Campus, Sejong City, Republic of Korea; 7 Interdisciplinary Graduate Program for Artificial Intelligence Smart Convergence Technology, Korea University, Sejong, Republic of Korea; 8 Jeonju Center, Korea Basic Science Institute (KBSI), Analysis & Researcher Division, Jeollabuk-do, Republic of Korea; 9 Department of Chemistry, Pukyong National University 45 Yongso-Ro, Nam-gu, Busan, Republic of Korea; 10 Department of Physics, Chungbuk National University, Cheongju, Republic of Korea; 11 Surface Materials Division, Korea Institute of Materials Science (KIMS), Changwon, Republic of Korea

**Keywords:** Displays, Organic LEDs

Correction to: *Nature Communications* 10.1038/s41467-021-23203-y, published online 17 May 2021.

The original version of this Article contained an error in page 6 line 13 which incorrectly read ‘However, the heat conductance (along the direction normal to the substrate) is dependent on the relative substrate thickness, which for thin NOA63 and the thick glass substrate are 543.38 and 37.5 [W K^−1^]’. The correct version states ‘0.543 and 0.0375 [W K^−1^]’. in place of ‘543.38 and 37.5 [W K^−1^]’.

This has been corrected in both the PDF and HTML versions of the Article.

The original version of the Supplementary Information associated with this Article contained an error in page 6 Supplementary Fig. 12, last row of the table, which incorrectly read ‘Thermal conductance P [W/K] 37.5, 543.48, 71.43, 23.81’. The correct version states ‘0.0375, 0.5434, 0.0714, 0.0238’ in place of ‘37.5, 543.48, 71.43, 23.81’.

The HTML has been updated to include a corrected version of the Supplementary Information.

## Supplementary information

Updated Supplementary Information

