# Supplementary material for: Highly efficient, heat dissipating, stretchable organic light-emitting diodes based on a MoO3/Au/MoO3 electrode with encapsulation
Source: Nat Commun. 2021 May 17;12:2864. doi: 10.1038/s41467-021-23203-y (PMC8128878; doi:10.1038/s41467-021-23203-y)
Supplement: Supplementary file 1 — Supplementary Information [file 41467_2021_23203_MOESM1_ESM.pdf]

Supplementary Information

Highly Efficient, Heat Dissipating, Stretchable Organic Light-Emitting Diodes Based on a MoO_3/Au/MoO_3 Electrode with Encapsulation

Dae Keun Choi[1,2,3,a], Dong Hyun Kim[1,2,3,a], Chang Min Lee[1,2,3,a], Hassan Hafeez[1,2,*], Subrata Sarker[1,2,3], Jun Su Yang[1], Hyung Ju Chae[1,2,3], Geon-Woo Jeong[1,2,3], Dong Hyun Choi[1,2,3], Tae Wook Kim[1,2,3], Seunghyup Yoo[4], Jinouk Song[4], Boo Soo Ma[5], Taek-Soo Kim[5], Chul Hoon Kim[6], Hyun Jae Lee[6], Jae Woo Lee[7], Donghyun Kim[7], Tae-Sung Bae[8], Seung Min Yu[8], Yong-Cheol Kang[9], Juyun Park[9], Kyoung-Ho Kim[10], Muhammad Sujak[10], Myungkwan Song[11], Chang-Su Kim[11,*], and Seung Yoon Ryu[1,2,3,*]

[1]*Division of Display and Semiconductor Physics, Display Convergence, College of Science and Technology, Korea University Sejong Campus, 2511 Sejong-ro, Sejong City 30019, Republic of Korea*

[2]*Department of Applied Physics, Korea University Sejong Campus, 2511 Sejong-ro, Sejong City 30019, Republic of Korea*

[3]*E-ICT–Culture-Sports Convergence Track, Korea University Sejong Campus, 2511 Sejong-ro, Sejong City 30019, Republic of Korea*

[4]*Department of Electrical Engineering, Korea Advanced Institute of Science and Technology (KAIST), Daejeon 34141, Republic of Korea*

[5]*Department of Mechanical Engineering, Korea Advanced Institute of Science and Technology (KAIST), Daejeon 34141, Republic of Korea*

[6]*Department of Advanced Materials Chemistry, College of Science and Technology, Korea University Sejong Campus, 2511 Sejong-ro, Sejong City, 30019, Republic of Korea*

[7]*Interdisciplinary Graduate Program for Artificial Intelligence Smart Convergence Technology, Korea University, Sejong 30019, Republic of Korea*

[8]*Jeonju Center, Korea Basic Science Institute (KBSI), Analysis & Researcher Division, 20 Geonji-ro, Deokjin-gu, Jeonju-si, Jeollabuk-do, 54907, Republic of Korea*

[9]*Department of Chemistry, Pukyong National University 45 Yongso-Ro, Nam-gu, Busan 48513, Republic of Korea*

[10]*Department of Physics, Chungbuk National University, Cheongju 28644, Republic of Korea*

[11]*Advanced Nano-Surface Department, Korea Institute of Materials Science (KIMS), Changwon, 51508, Republic of Korea*

[a]These authors equally contributed to this paper.

[*]Corresponding author List

Prof. Dr. Seung Yoon Ryu[1,2,3,*]

[1]Division of Display and Semiconductor Physics, College of Science and Technology, Korea University Sejong Campus, 2511 Sejong-ro, Sejong City 30019, Republic of Korea;

[2]Department of Applied Physics, Korea University Sejong Campus, 2511 Sejong-ro, Sejong City 30019, Republic of Korea;

[3]E-ICT–Culture-Sports Convergence Track, Korea University Sejong Campus, 2511 Sejong-ro, Sejong City 30019, Republic of Korea,

Tel) +82-44-860-1376, justie74@korea.ac.kr

Dr. Chang-Su Kim[11,*]

[11]Advanced Nano-Surface Department, Korea Institute of Materials Science (KIMS) Changwon, 51508, Republic of Korea,

Tel)+82-55-280-3696, cskim1025@kims.re.kr

Dr. Hassan Hafeez[1,2,*]

[1]Division of Display and Semiconductor Physics, College of Science and Technology, Korea University Sejong Campus, 2511 Sejong-ro, Sejong City 30019, Republic of Korea,

Tel) +82-44-860-1376, hassaniskt@hotmail.com

Keywords: Twistable GSOLEDs (Geometrically Stretchable Organic Light-Emitting Diodes), Ultra Violet (UV)-curable polymer (NOA63), Water-Proof Encapsulation, Light Scattering, Silicon Dioxide (SiO_2) Nano Particles (NPs), Heat dissipation, Mechanical Neutral Plane (MNP), Efficiency Roll-Off

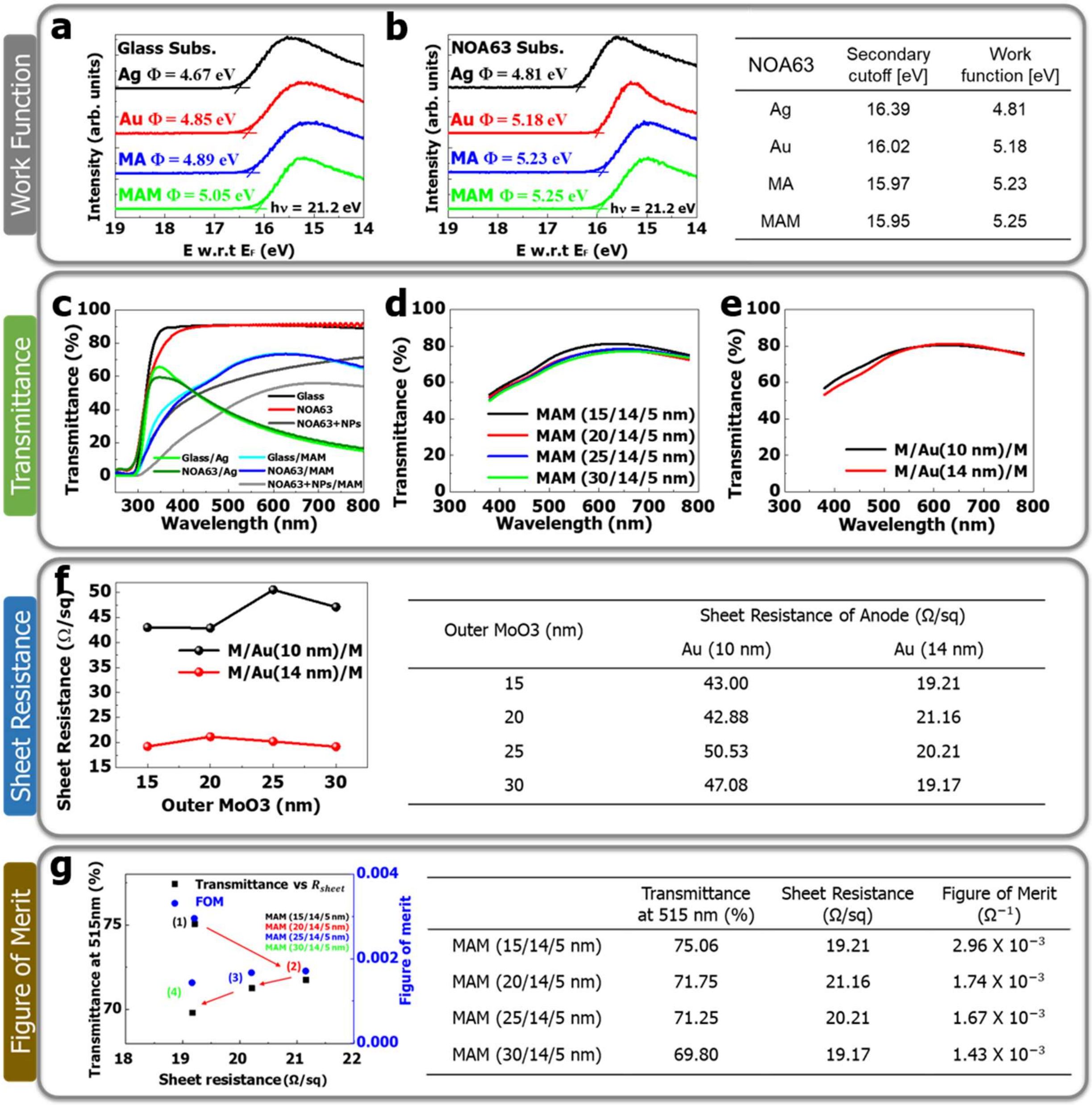

Figure 1. Optical and electrical properties analysis of the respective layers. **a-b,** The ultraviolet photoelectron spectroscopy (UPS) analysis in both cases between glass and Norland optical adhesive (NOA63) substrate presented a similar trend with a small difference in values. We speculate that the surface roughness of the substrate or exposure to the environment might have affected the UPS measurement because the work function (φ) values were slightly deviated. Interestingly, when an outer molybdenum trioxide (MoO_3) layer (15 nm) was added

before the deposition of Au (i.e. MA), the φ was increased to ~5.23 eV due to Fermi-Level pinning and the higher φ of the MoO_3 layer (~6.6 eV[1, 2]). The φ of the electrode was further enhanced to ~5.25 eV by the deposition of an inner MoO_3 (5 nm) in addition to the MA structure (i.e. MAM) and is more beneficial for device performance, hence, improving the electrical and optical properties.[3, 4, 5] **c,** The glass and NOA63 demonstrated an almost equally high transmittance of about 90% due to the intrinsic properties. When the Ag was deposited on both substrates, the transmittance considerably dropped to about 60% at ~400 nm and to ~23%,[6] at ~700 nm, which induces the microcavity effect in the organic light emitting-diodes (OLEDs) devices due to the high reflection as reported by Jalil et al.[7] However, when a MAM electrode was deposited on either NOA63 or the glass substrate, a higher transmittance was obtained that was ~45% and ~70% at about 400 and 650 nm, respectively. Here, the transmittance of the NOA63 with nanoparticles (NPs) was also found to be ~20% less than NOA63 without NPs, which was offset by the ~20-30% haze effect (discussed later in Supplementary Figure 14). **d,** In the MAM aspect, Hong et. al.[8] reported that by optimizing the thickness of the outer oxide layer, the surface plasmon generated by the sandwiched metal could be coupled with the air using Bragg's scattering, thus increasing the overall transmittance. **e-f,** The thickness of the sandwiched metal layer is also crucial as it causes a trade-off between transmittance and conductivity as reported by Wrzesniewski et al.[9] It was observed that the 14 nm Au layer presented a very low sheet resistance of ~19.2 Ω/sq, which made it the perfect candidate to be used in the MAM electrode structure. **g,** MoO_3(15 nm)/Au(14 nm)/MoO_3(5 nm) demonstrated the best figure of merit ($\emptyset_{TCO(1)}$)[10, 11] value (2.96 × 10^{-3} Ω-1),

$$\emptyset_{TCO(1)} = \frac{T^{10}}{R_{\text{sheet}}} = \frac{(0.7506)^{10}}{19.21} \approx 2.96 \times 10^{-3}[\Omega^{-1}] , \quad (1)$$

where T is the transmittance and R_{sheet} is the sheet resistance. The figure of merit value suggest a fair balance or an optimized performance, between a high transmittance (70~80%) and a low sheet resistance (~ 20 Ω/sq).

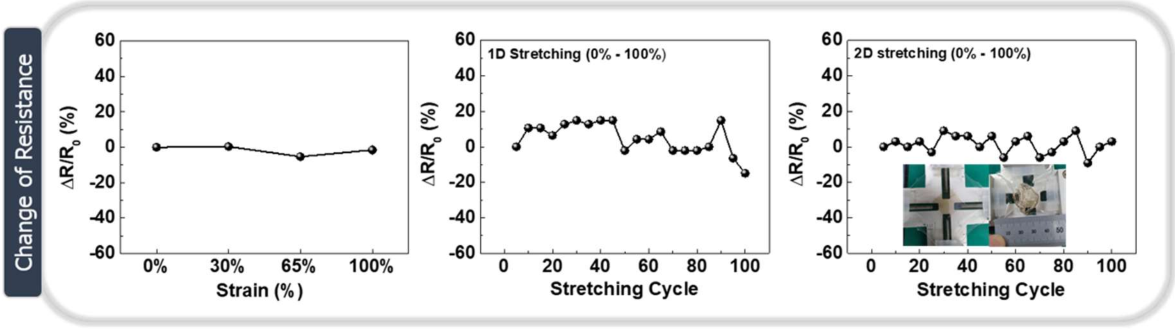

Transparent Electrodes	Advantages	Disadvantages	Ref
PEDOT:PSS	• High transmittance (>80%)<br>• Solution processable<br>• Well matched for Hole injection material<br>• Suitable for stretchable electronics (flexible)	• Poor environmental stability<br>• ITO etched by acidic PEDOT:PSS<br>• High sheet resistance	[12]
ITO	• High transmittance (>90%)<br>• Low sheet resistance (<20 Ohm⁄sq)<br>• Well matched for Hole injection electrode	• Poor mechanical flexibility thus not suitable for stretchable electronics<br>• High deposition temperature (>300 °C)<br>• Increasing cost of indium	[13]
AgNWs	• Suitable for intrinsically, geometrically stretchabl e electronics<br>• High transmittance (>80%)<br>• Low sheet resistance (10 to 50 Ohm⁄sq)<br>• Good mechanical flexibility	• Rough surface<br>• Poor environmental stability (native oxide)<br>• Network meltdown into nanoparticles	[14]
MAM [MoO_3 (15nm) / Au (14nm) / MoO_3 (5nm)]	• Low deposition temperature (<300 °C)<br>• High transmittance (>80%)<br>• Low sheet resistance (10 to 50 Ohm⁄sq)<br>• High environmental stability<br>• Suitable for geometrically stretchable devices<br>• Mechanical ductility<br>• High work function (~5.25 eV, MAM)	• Not suitable for intrinsically stretchable, suit able geometrically stretchable electronics<br>• Water soluble material (MoO_3)<br>• Expensive material (Au)	[1]<br>[3]

Figure 2. Advantages and disadvantages of transparent electrodes: PEDOT:PSS[12], ITO[13], AgNWs[14] and MAM[3]. The key advantages of MAM over other electrodes are high transmittance, low sheet resistance, and high environmental stability. Also, MAM electrode has good mechanical flexibility as the rate of resistance change of the electrode remained almost unchanged with 1D and 2D stretching cycles.

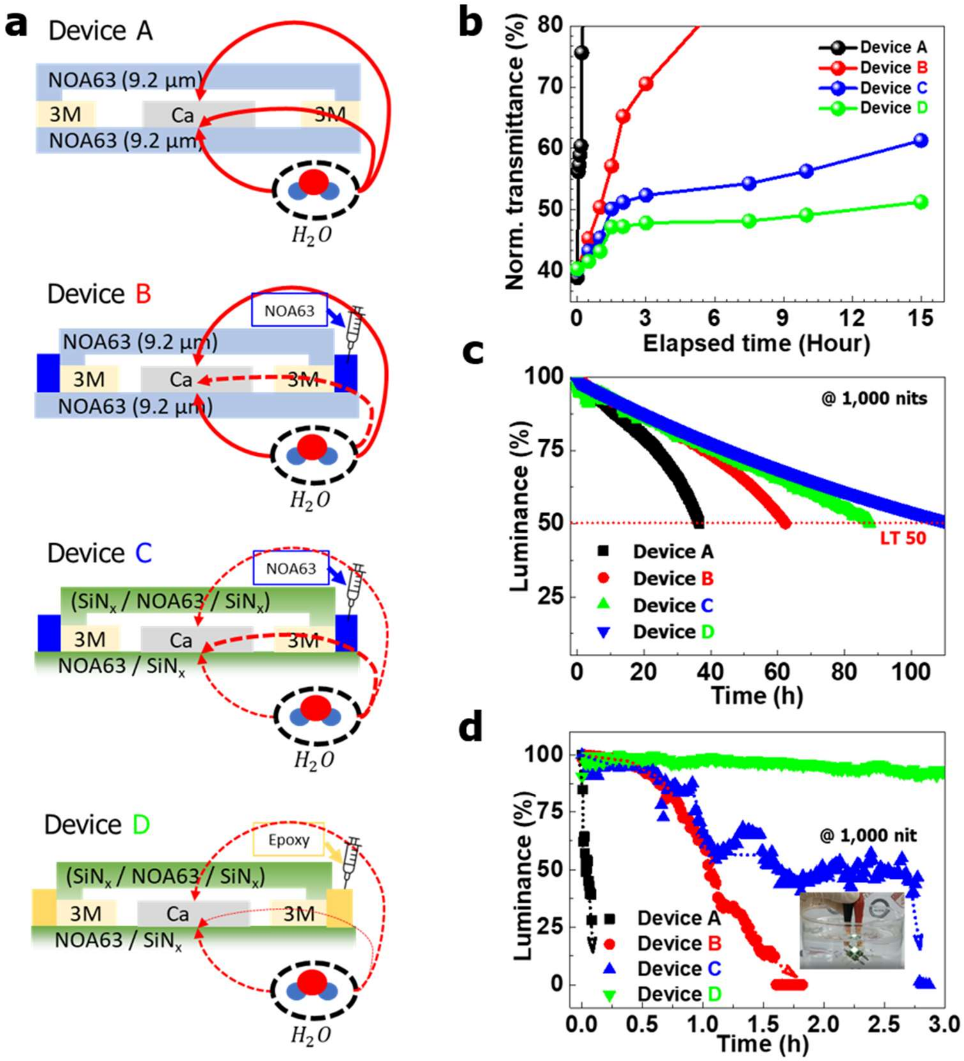

Encapsulation	Device A	Device B	Device C	Device D
WVTR ($gm^{-2}day^{-1}$)	4×10^{0}	2.0×10^{-1}	3.6×10^{-2}	1.8×10^{-2}

Figure 3. Water vapor transmission rate (WVTR)[15, 16, 17] comparison among different encapsulations and device lifetime of the thick 3M elastomer based GSOLED devices with various encapsulations. **a,b** The NOA63 and NOA63 (9.2 μm)/SiN_x (200 nm) substrates were coated with calcium (Ca) to understand the moisture absorption mechanism of the material when encapsulated with different encapsulation materials (Device A : NOA63/only 3M tape side passivation/NOA63; Device B : NOA63/3M tape and NOA63 side passivation/NOA63; Device C : NOA63/SiN_x/3M tape and NOA63 side passivation/SiN_x/NOA63/SiN_x;

Device D : NOA63/SiN_x/3M tape and epoxy side passivation/SiN_x/NOA63/SiN_x). Ca was deposited with a shadow mask in the shape of N × N array of patches, which is suitable for evaluating the improvement of the gas barrier film, because with a large single Ca patch it is difficult to determine whether the pinhole is suppressed. The moisture absorbed by the Ca was analyzed using the WVTR equation given as[15, 16, 17]:

$$WVTR = \rho(\mathrm{CaO}) \times \frac{m(\mathrm{H_2O})}{m(\mathrm{CaO})} \times \frac{D(\mathrm{CaO})}{t} \ : \ [\frac{\mathrm{g}}{\mathrm{m^2 day}}], \quad (2)$$

where ρ is the density, D is the thickness of the CaO layer which changes with the absorption of moisture, m is the molar concentration, and t is the time passed. The values of ρ and m of water and CaO are known, thus Equation (2) becomes:

$$WVTR = 3.35 \times \frac{10^6\mathrm{g}}{m^3} \times \frac{18.0152\ \mathrm{g/mol}}{56.077\ \mathrm{g/mol}} \times \frac{D(\mathrm{CaO})}{t} \ : \ [\frac{\mathrm{g}}{\mathrm{m^2 day}}] \quad (3)$$

Here, D can be analyzed by measuring the transmittance of the layer, which increases with the passage of time due to moisture absorption and is given by:

$$D(\mathrm{CaO}) \sim \frac{\mathrm{Final\ transmittance} - \mathrm{Initial\ transmittance}}{100\ \% - \mathrm{Initial\ transmittance}} \times \mathrm{deposited\ thickness} \quad (4)$$

The deposited thickness was ~$500 \times 10^{-10} m$. We analyzed the transmittance values of the devices with different substrates and encapsulations and put them to Equation (4). The values of WVTR obtained for the devices are summarized in the table of Supplementary Figure 3. The device A showed the highest WVTR of 4×10^0 g m^{-2} day^{-1} and the WVTR value of device B was improved when 3M tape was used together with NOA63 at the side passivation (2.0×10^{-1} g m^{-2} day^{-1}). We speculate that the 3M tape alone as a sealant was not the proper material to use for the purpose, as it could have allowed moisture to penetrate into the substrate. Comparing the WVTR of device C (3.6×10^{-2} g m^{-2} day^{-1}) and device D (1.8×10^{-2} g m^{-2} day^{-1}), epoxy was found to be better in blocking the moisture at side than NOA63. Thus, it may be concluded that the side encapsulation is much more important than the penetration from top and bottom films. From the above comparisons, we identified the encapsulation materials in three different paths – bottom (substrate), top (encapsulation), and side (sealant) – in the order of decreasing ease of moisture penetration as red solid lines, and red dot lines. Even though the encapsulations (device B, C and D) suggested improved WVTR than that of device A, those devices were not fully stretchable. There is a trade-off relation between encapsulation

quality and stretchability. Therefore, it needs further study to implement edge encapsulation using stretchable sealant for GSOLEDs. **c,d**, As suggested in the Figure 2, the device lifetime (LT) under various encapsulation schemes and water immersion condition have improved and were comparable, respectively (shown in Supplementary Figures 3c, d). All of data trend was found to be coherent with the conditions. It may be concluded that the "side passivation" from a combination of 3M tape and NOA63 is quite critical as well as protective against "film penetration" through NOA63 films in a vertical direction. Device A encapsulation withstood for about 7 min during the water immersion, while Device B and C with NOA63 side passivation survived for over 1~3 hours. The shorter LT0 of the device A encapsulation as compared to its LT50 (Supplementary Figures 3c, d) is due to side penetration of water, which was confirmed by WVTR measurement (Supplementary Figure 3b). Even though the side encapsulation with epoxy (Device D) was quite effective, there is a trade-off between stretchability and encapsulation. It needs further study to improve the stretchable encapsulation for GSOLEDs.

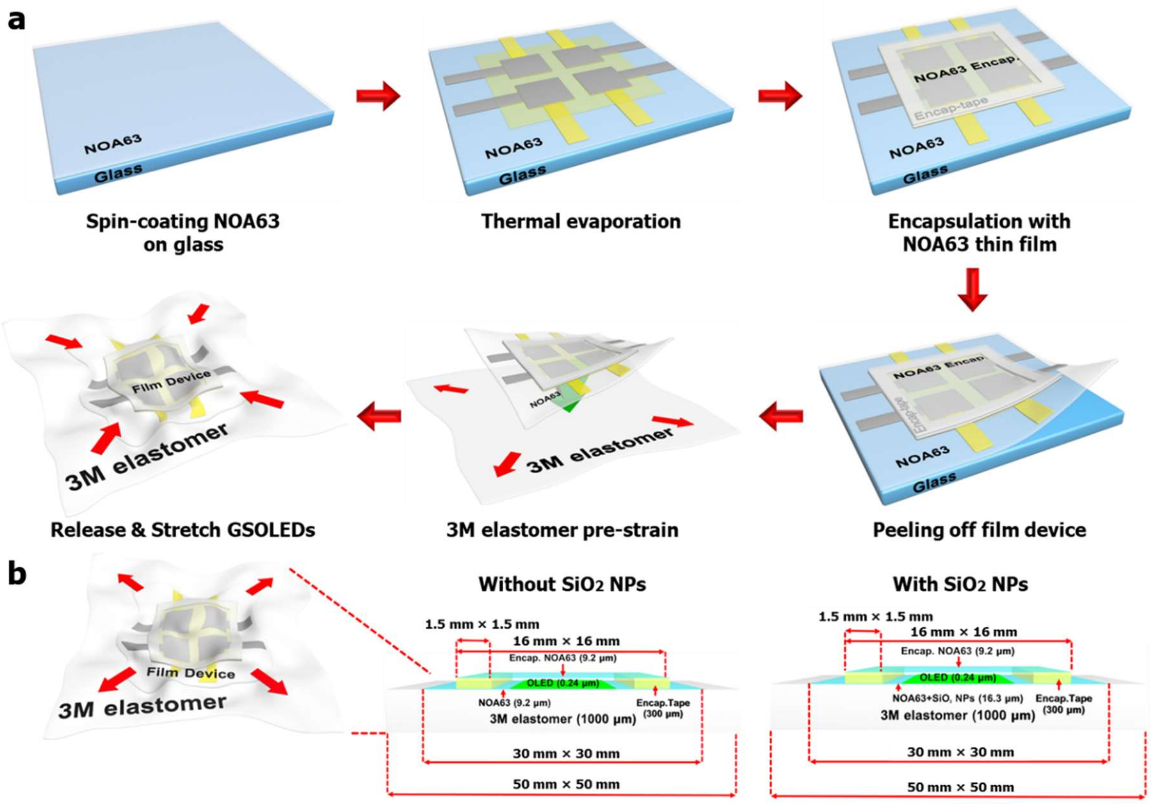

Figure 4. Schematic illustration of the device fabrication by kinetic transfer printing along with dimensions. **a,** The NOA63 with and without silicon dioxide (SiO_2, 470 nm sized) NPs was spin coated on a glass substrate to form a thin layer of ~16.3 and ~9.2 μm thickness, respectively. The OLEDs constituent layers were deposited on the NOA63 using thermal evaporation followed by NOA63 encapsulation (~9.2 μm). The thin substrate and encapsulation ensured the formation of a sandwich structure that could compensate for the small bending strain and the adjustment of mechanical neutral plane (MNP) at the high Young's modulus (YM) layers of the device.[18] Thus, the crucial layers responsible for the performance of the OLED were preserved from any compression or tensile stresses during stretch-release or bending cycles. The encapsulation also ensured the device was moisture resistant and aided the performance of the GSOLEDs during water-immersion (Fig. 2c and Supplementary Figure 3). **b,** The schematic illustration of the side view for the GSOLED device demonstrates the thickness of the layers and the overall layout with and without NPs. The release of pre-strain resulted in the formation of buckles in the devices shown in the image. The NOA63 encapsulation was performed with a small N_2/Air gap thus forming a protective cover from moisture contents enabling the device to be water-proof.

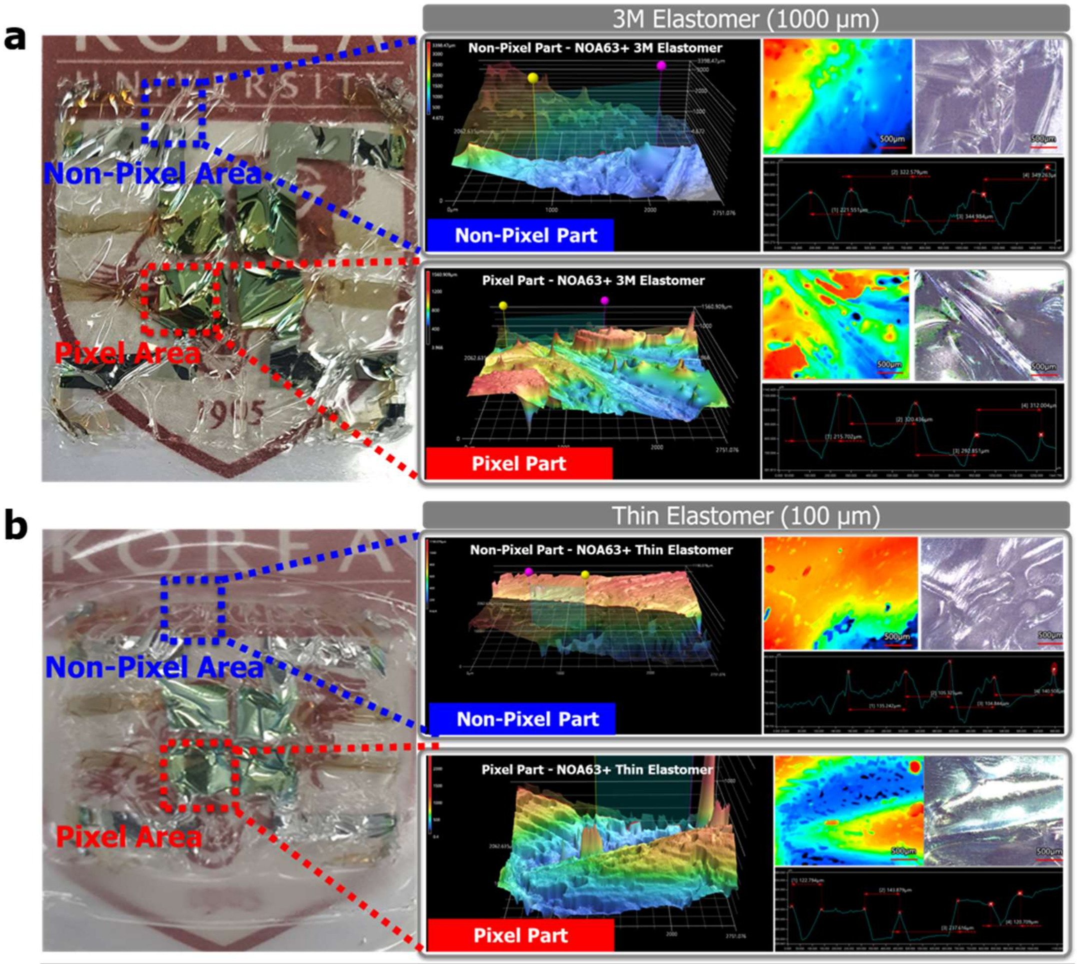

Peak to Peak of Buckle Formation

Part		1st	2nd	3rd	4th	Avg.
3M Elastomer (1000μm)	Pixel Area	216 μm	320 μm	293 μm	312 μm	280±47 μm
	Non-Pixel Area	221 μm	322 μm	345 μm	349 μm	309±60 μm
Thin Elastomer (100μm)	Pixel Area	123 μm	144 μm	237 μm	121 μm	156±47 μm
	Non-Pixel Area	135 μm	105 μm	105 μm	140 μm	121±19 μm

Figure 5. Camera and confocal microscopy (CM) images of pixel and non-pixel area in the GSOLEDs. The optical and CM images of the GSOLEDs based on **a,** 3M elastomer and **b,** thin elastomer showed that there were as much buckling on the pixel area as on the non-pixel area. Average buckling periodicity on the pixel area of 3M elastomer was 280 μm, whereas buckling on the non-pixel area was 309 μm. When thin elastomer was used, the buckling periodicity on pixel and non-pixel area was 156 μm and 121 μm, respectively. Thus, thinner elastomer is helpful to make smaller buckling periodicity, however, we are limited by the current strain threshold of the thin elastomer materials for the realization of small buckling devices.

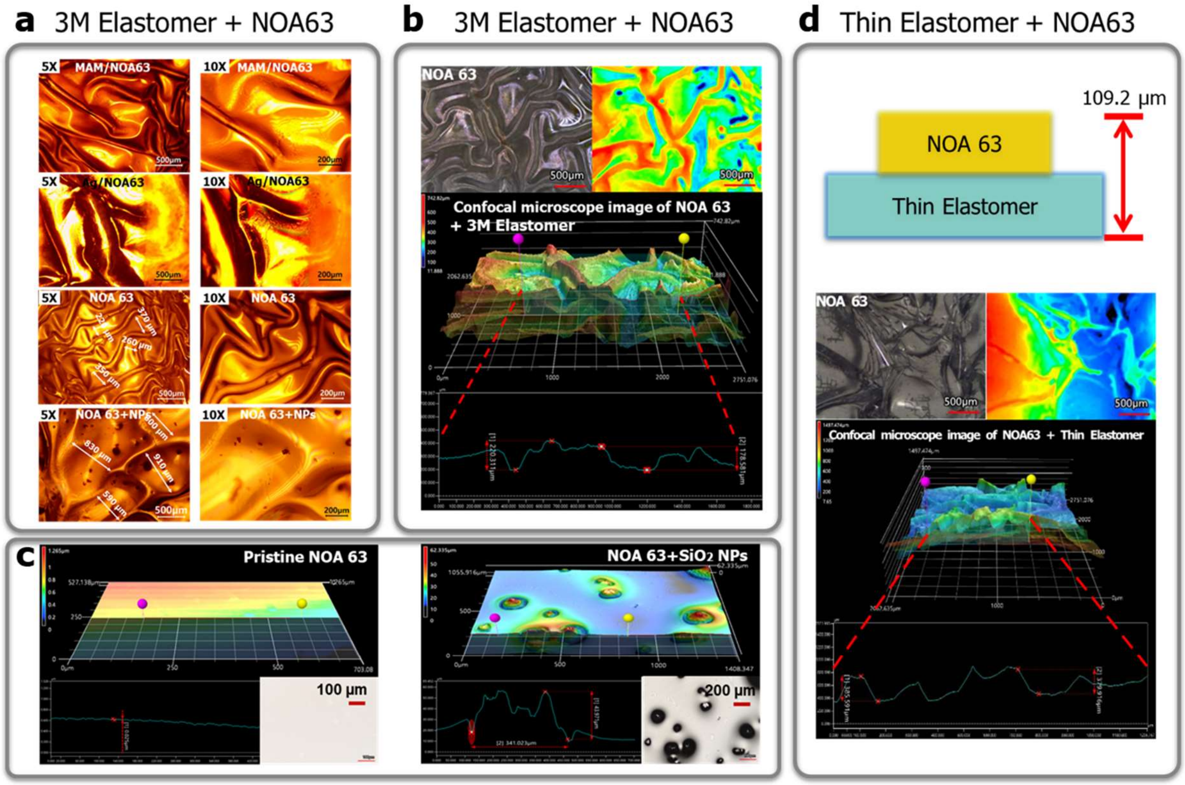

Peak to Peak of buckle formation<br>Substrates	1st	2nd	3rd	4th	Avg.
3M Elastomer + NOA63	220 µm	260 µm	350 µm	370 µm	300±72 µm
3M Elastomer + NOA63 + NPs	590 µm	800 µm	830 µm	910 µm	783±136 µm
Thin Elastomer +NOA63	204 µm	102 µm	182 µm	132 µm	155±53 µm

Figure 6. Optical microscope (OM) images of the electrodes and NOA63 at 5× and 10× magnification along with confocal microscopy (CM) images. **a,** The OM images of the MAM electrodes at 5× and 10× magnification present the surface morphology after removal of pre-strain. It was observed that the random buckles were formed due to the contraction stresses in the layers. On average the size of the buckles could be estimated to be ~100 µm at maximum and they were closely packed (i.e. high density/number). The buckles formed in the Ag electrode layer were of much larger dimension. This could have occurred because the Ag might have poorly adhered to the NOA63 substrate, causing random parts of the Ag layer to delaminate. This factor affected the performance of the devices because the efficiency had deteriorated. OM images of NOA63 demonstrate that the buckles were of much smaller dimensions, which indicates that the polymer layer had the lowest resistance to the induced stresses. Short wavelength (NOA63 without NPs) and long wavelength (NOA63 with NPs) buckles acted as

the main driving force for various complex mechanics of the devices including stretching, folding, twisting and water-proofing. **b,** The NOA63 buckled substrate was also analyzed using CM images for the dimensions of the peaks and valleys. The analysis was found to be in agreement with the OM analysis. The maximum height of the buckles from the valley was found to be ~220 µm while from the flat area it was ~178 µm, therefore, the average size of the fabricated buckles could be analyzed to be 200 ± 21 µm. With these dimensions, the substrates were conveniently stretched up to 100% area (2D) and were found to be reasonable for stretchable applications. **c,** It could be observed that the top surface of NOA63 without NPs demonstrate a somewhat smooth surface, however, some bumps were observed in ultra-high resolution field emission scanning electron microscopy (UHR FE-SEM) (Supplementary Figure 8c) and CM images. The dimensions of these bumps, measured through CM imaging, were ~0.4 µm (400 nm). The CM images for the top surface of NOA63 with NPs, present some three-dimensional optical sectioning representing the changes in the morphology of the surface. **d,** More small buckling periodicity (average value, ~155 µm) occurred in thin elastomer (100 µm), while large buckling periodicity (average value, ~300 µm) in thick elastomer (1,000 µm) was observed. It was reported that the buckling periodicity depends on both polymer substrates and elastomer; that means, the overall thickness of the device including substrate and elastomer determines the periodicity of wrinkled structure.[19, 20, 21] Thus, we found that there were two important factors for micro buckling formation; one is the thickness of substrate and the other one is the thickness of elastomer.

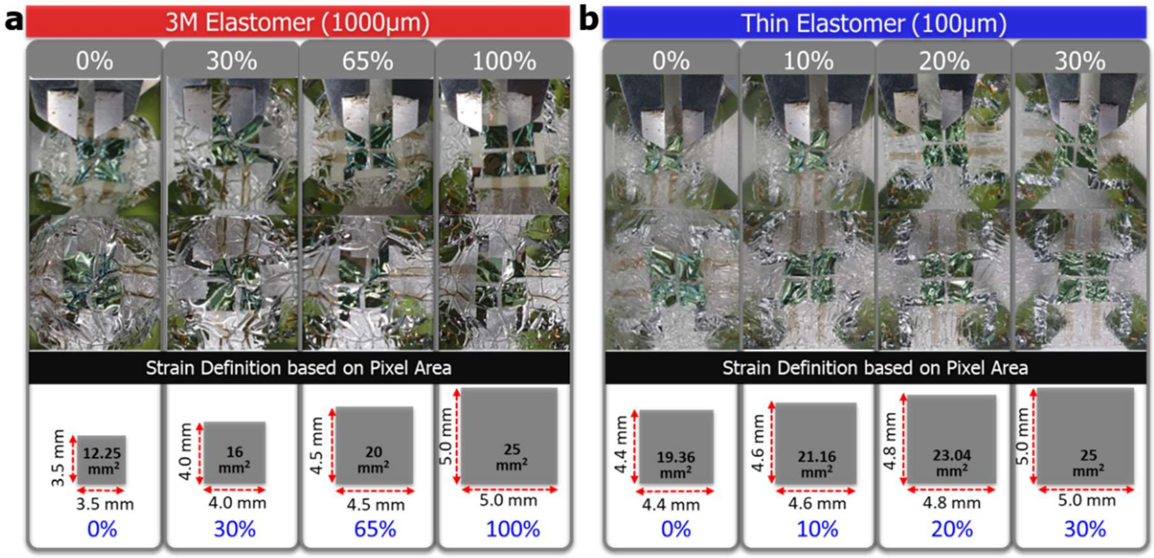

	Pre-Strain (%)	Initial Pixel Area (mm^2)	Strained Pixel Area (mm^2)	Changed Pixel Area (mm^2)	2D Stretched (%)
3M Elastomer (1000μm)	100	12.25	12.25	0.00	0
			16.00	3.75	30
			20.25	8.00	65
			25.00	12.75	100
Thin Elastomer (100μm)	30	19.27	19.36	0.00	0
			21.16	1.92	10
			23.04	3.85	20
			25.00	5.73	30

Figure 7. Camera images and schematic illustration of the pixel area under the different strain on the GSOLEDs. The camera images of buckled pixel area with **a,** the 3M elastomer and **b,** thin elastomer. For the complete comparison, we have added the schematic illustration below the camera images. At first, we transferred the OLED devices on pre-strained elastomers (3M elastomer and thin elastomer) until their limit (100% and 30%). When we released the strain from the elastomer as shown in Supplementary Figure 4, the pixel areas (5 mm × 5 mm) shrunk to 3.5 mm × 3.5 mm (3M elastomer) and 4.4 mm × 4.4 mm (thin elastomer) according to the initial sizes of the elastomers. Thus, the 2D strain in the GSOLEDs can be defined as percentage of the initially deformed pixel area (A_i) and deformed pixel area (A_d) with respect to the non-deformed pixel area (A_{nd} = 25 mm^2) that is, strain = [($A_{nd} - A_d$) / A_i] × 100%. i.e. 65 % strain = [(25 mm^2 – 16 mm^2) / 12.25 mm^2] × 100% at the 3M elastomer.

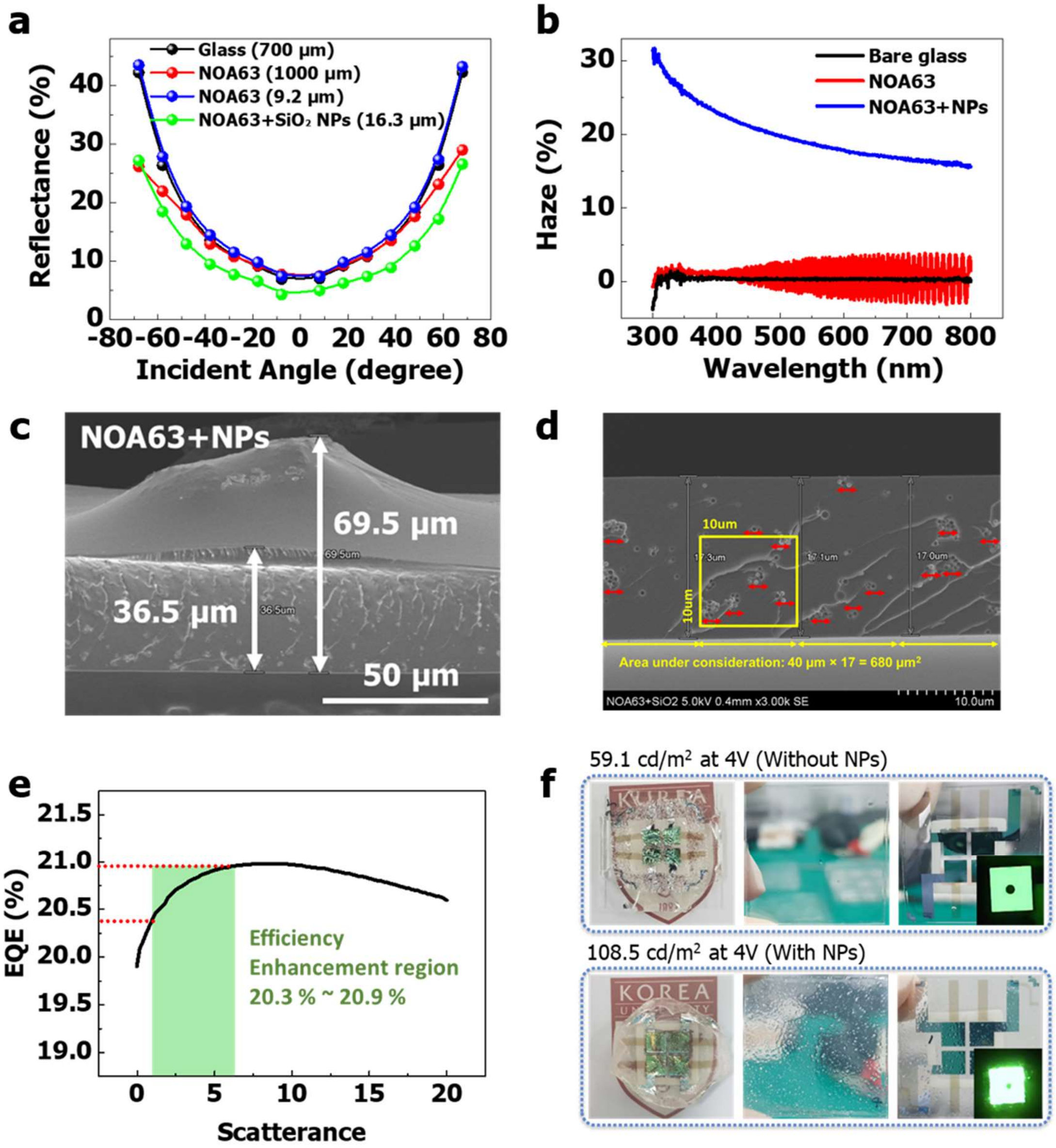

Figure 8. Reflectance, haze analysis and scattering properties of SiO_2 NPs in NOA63. **a, b,** A decrease in reflectance was observed for the SiO_2 NPs incorporated NOA63 (~5% at 0° and ~27% at 60°) as compared to pristine NOA63 (~8% at 0° and 42% at 60°). This decrease in reflectance of NOA63 with NPs was compensated for by the high haze (~20% at 510 nm) indicating a forward Mie scattering effect[22, 23] by the NPs. **c, d,** The cross-sectional FE-SEM images to show the thickness and NPs distribution in the NOA63 layer. There was a large thickness deviation of NOA63 with NPs, from 16.3 ~ 69.5 μm because the NPs are not uniformly distributed inside NOA63 due

to the surface modification and agglomeration of NPs by the UV treatment.[24, 25] **e,** The calculation on mean free path and scatterance by NOA63 thickness dependence.

$$\text{Mean free path} = \frac{1}{\left\{\left(\frac{25}{1000\ \mu\text{m}^3}\right)\times 3.14\ \mu\text{m}^2\right\}} = 12.74\ \mu\text{m} \qquad (5)$$

$$S = \frac{d}{\text{Mean free path}}, \qquad (6)$$

where S is the scatterance and d is the thickness of NOA63; e.g., when d is 69.5 µm, the S is 5.45. The calculated S was observed around EQE 20.3% ~ 20.9%, which means NOA63 with 470 nm SiO_2 NPs effectively enhanced optical efficiency. **f,** The comparison of the buckled image with and without NPs in the GSOLED combining the NOA63 film on glass substrate and the pixel image. The NOA63 without NPs presented a clear and transparent view but did not show any scattering effect or increased brightness, while the NOA63 with NPs demonstrates a hazy (opaque) view but presents a much higher brightness.

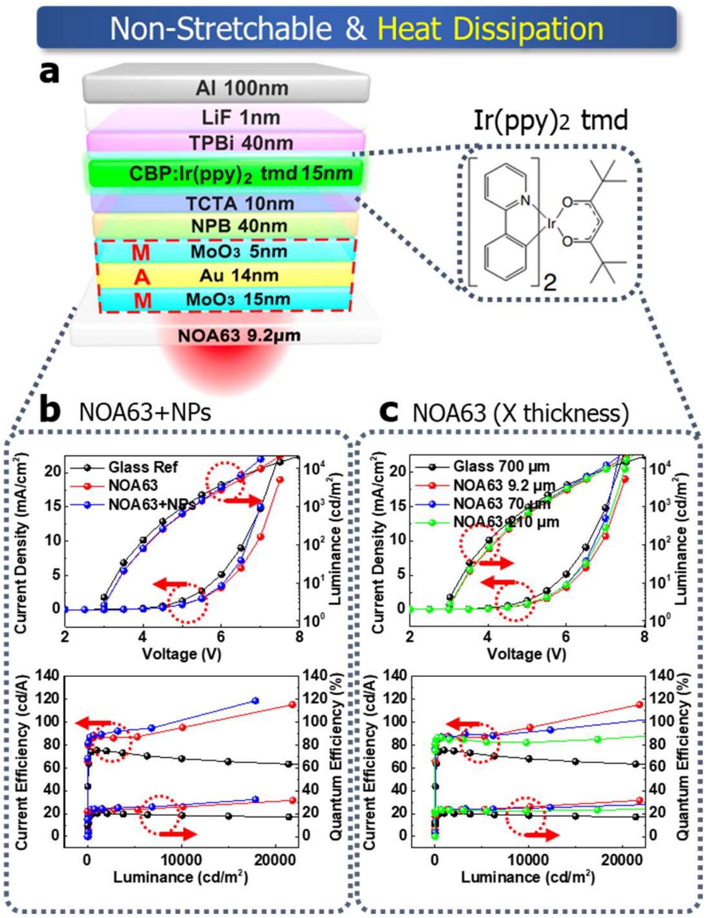

Figure 9. The performance of the heat dissipating devices with horizontal emitters and different thicknesses of elastomers with and without NPs. **a,** the schematic illustration of non-stretchable and heat dissipating device. **b, c,** The performances, such as current injection, luminance, and various efficiencies improved as the thickness of the elastomer (NOA63) was decreased due to improved heat dissipation. Moreover, addition of NPs to the NOA63 film improved the device efficiency due to increased out-coupling. Interestingly, the glass-based device (700 μm) presented the efficiency roll-off as the luminance was increased, while thin NOA63 based devices displayed slightly increased efficiency without the efficiency roll-off over 100 cd/A and EQE 20% at 20,000 nits. To explain this phenomenon, the temperature of the thin NOA63 and thick glass substrate surface was directly measured by an infrared (IR)-camera as shown in Fig. 5b and Supplementary Figure 13.

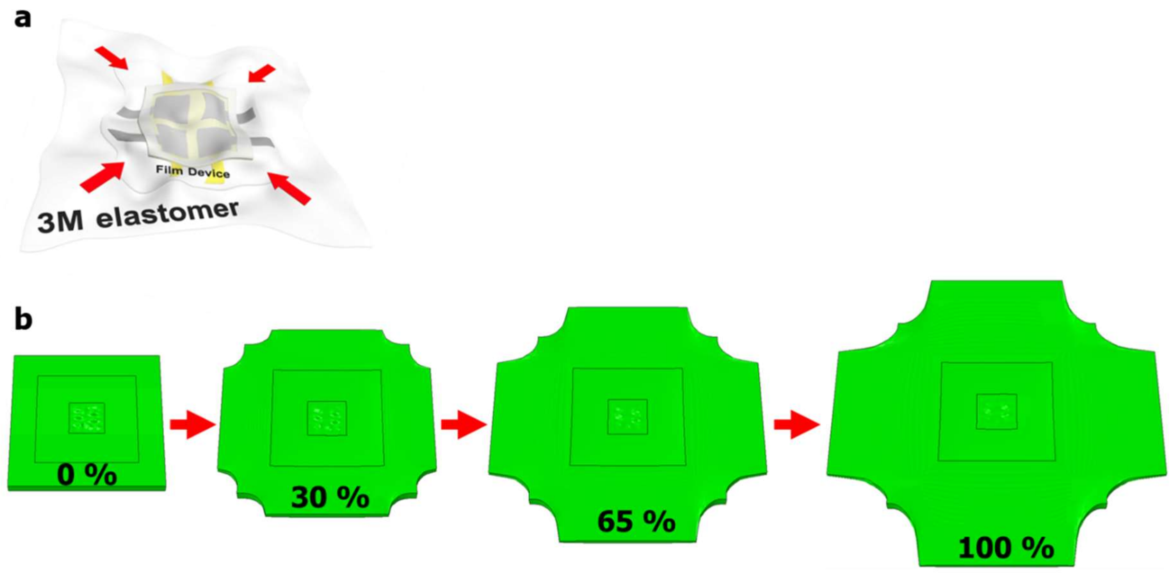

Figure 10. Mechanical simulation and governing equation. **a,** The schematic illustration of the GSOLEDs on the 3M elastomer. **b,** Governing equation of linear buckling analysis was used in FE simulation.

$$(K_0^{NM} + \lambda_i K_\Delta^{NM})\mathbf{v}_i^M = 0, \qquad (7)$$

where K_0^{NM}, K_Δ^{NM}, λ_i and $\mathbf{v}_i^M$and i are the initial stiffness matrix, differential load stiffness matrix, eigenvalues and buckling mode shape, respectively. *M* and *N* are the degree of freedom and *i* is i^{th} buckling mode. This means that they are not implemented in FE simulation using a static solver because there is no load or moment component that will cause bending in the lateral direction. Therefore, we used linear buckling analysis which can simulate wrinkled structures in the device.[26] First, the eigenvalue problem was solved in FE simulation with consideration of structure and mechanical properties of the device. The buckled shape of the devices was analyzed with a nontrivial solution obtained from the govern equation. Randomly wrinkled OLED was modelled by controlling displacement boundary conditions in the pre-stretch state.[27] Then, the biaxial stretching of 3M elastomer was simulated by importing the results of the buckling analysis. Simulation model for each strain (0%, 30%, 65%, and 100%) to reflect the real situation when the device is mounted and stretched on the stretching jig. We have evaluated the mechanical simulation for the stress on various strains (0%, 30%, 65%, and 100%) of the GSOLED with 3M elastomer and reflected boundary conditions of gripping edges as in actual experiment for stretching model to overcome the limitation of low convergence and the restriction of stretching model.

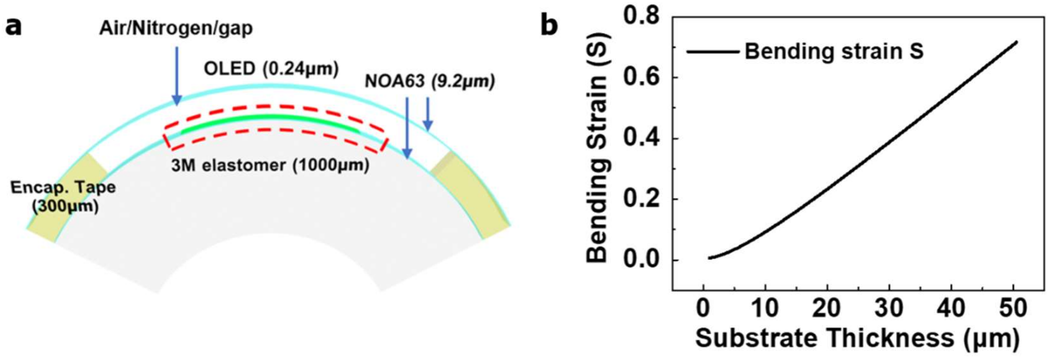

S	t_{OLED} (μm)	$t_{sub.}$ (μm)	R (μm)	χ	E_{OLED} (GPa)	$E_{sub.}$ (GPa)
0.005978	0.24	1	30	39.375	63	1.6
0.034219	0.24	5	30	39.375	63	1.6
0.091746	0.24	10	30	39.375	63	1.6
0.159828	0.24	15	30	39.375	63	1.6
0.233089	0.24	20	30	39.375	63	1.6
0.309273	0.24	25	30	39.375	63	1.6
0.387270	0.24	30	30	39.375	63	1.6
0.466467	0.24	35	30	39.375	63	1.6
0.546501	0.24	40	30	39.375	63	1.6
0.627140	0.24	45	30	39.375	63	1.6
0.708233	0.24	50	30	39.375	63	1.6

Figure 11. Bending strain calculation on thin NOA63 (9.2 μm) substrate. a, Shows the schematic of the device with encapsulation. **b,** The relationship between the bending strain and substrate thickness was plotted using the obtained data which indicates that the bending strain on the buckled devices would increase with an increase in substrate thickness. The bending strain on the devices was calculated using the physical properties of the substrate i.e, YM, thickness of the polymer substrate, and the overall OLED device thickness and YM parameters.

$$S = \frac{t_{OLED}+t_{sub}}{2R}\left[\frac{1+2\eta+\chi\eta^2}{(1+\eta)(1+\chi\eta)}\right], \quad (8)$$

where t_{OLED} and t_{sub} are thickness of OLEDs and substrate and R is the bending radius, respectively. And, η and χ are defined as t_{OLED}/t_{sub} and E_{OLED}/E_{sub}, where E_{OLED} and E_{sub} are Young's modulus of the OLED material and the polymer substrate. Young's modulus of 3M elastomer, NOA63, and OLED are defiend as 1.4 MPa, 1.6GPa, and 63 GPA, individually. **c,** The values for the thickness, YM and other parameters utilized for the bending strain calculation.

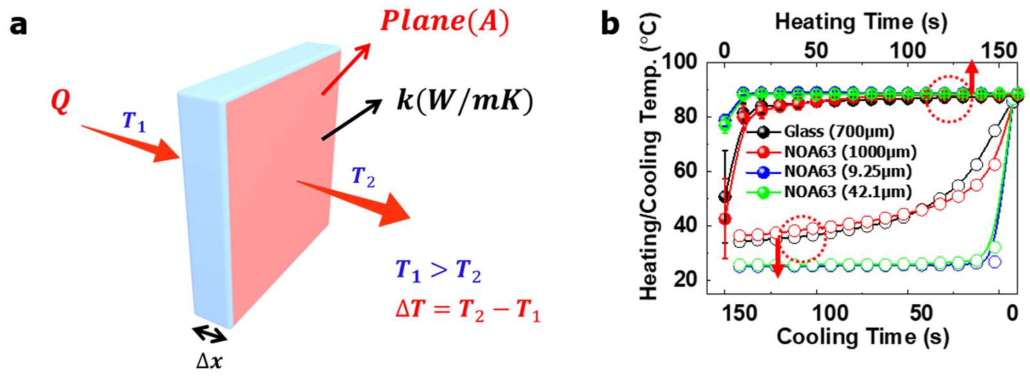

Substrates	Glass	NOA63	NOA63	NOA63
Δx (Thickness)	700 μm	9.2 μm	70 μm	210 μm
ΔT	-1	-1	-1	-1
A (Area)	25 mm^2	25 mm^2	25 mm^2	25 mm^2
Thermal conductivity $k\ [W/mK]$	1.05	0.2	0.2	0.2
Thermal conductance $P\ [W/K]$	0.0375	0.5434	0.0714	0.0238

Figure 12. The heat conductance, heating / cooling temperature for various substrates. a, The illustration for the concept of heat conductivity and conductance. The heat conductance (P) is the function of thickness and temperature as shown in the equation (9).

$$P = \frac{dQ}{dt} = -kA\frac{\Delta T}{\Delta x}, \qquad (9)$$

where Q, k, A, x and T are the transferred energy, heat conductivity, area, thickness and temperature, respectively. **b,** Heating and cooling temperature for glass substrate (700 µm), NOA63 substrate (9.2 µm, 1000 µm) and NOA63 with NPs (13.6 µm). In case of heating, the temperature of the thin NOA63 substrate (9.2 µm, 70 µm) quickly increased on the hot plate with time, while that of the thick NOA63 (1000 µm) and glass substrate (700 µm) slowly increased. In case of cooling, the thin substrate quickly decreased and thick substrate slowly decreased, due to the mechanisms of heat conductivity and conductance. **c,** The values of heat conductivity and conductance for glass substrate (700 µm) and NOA63 substrate (9.2 µm, 70 µm, 210 µm). It was calculated by the Poisson's equation for heat flow.[28]

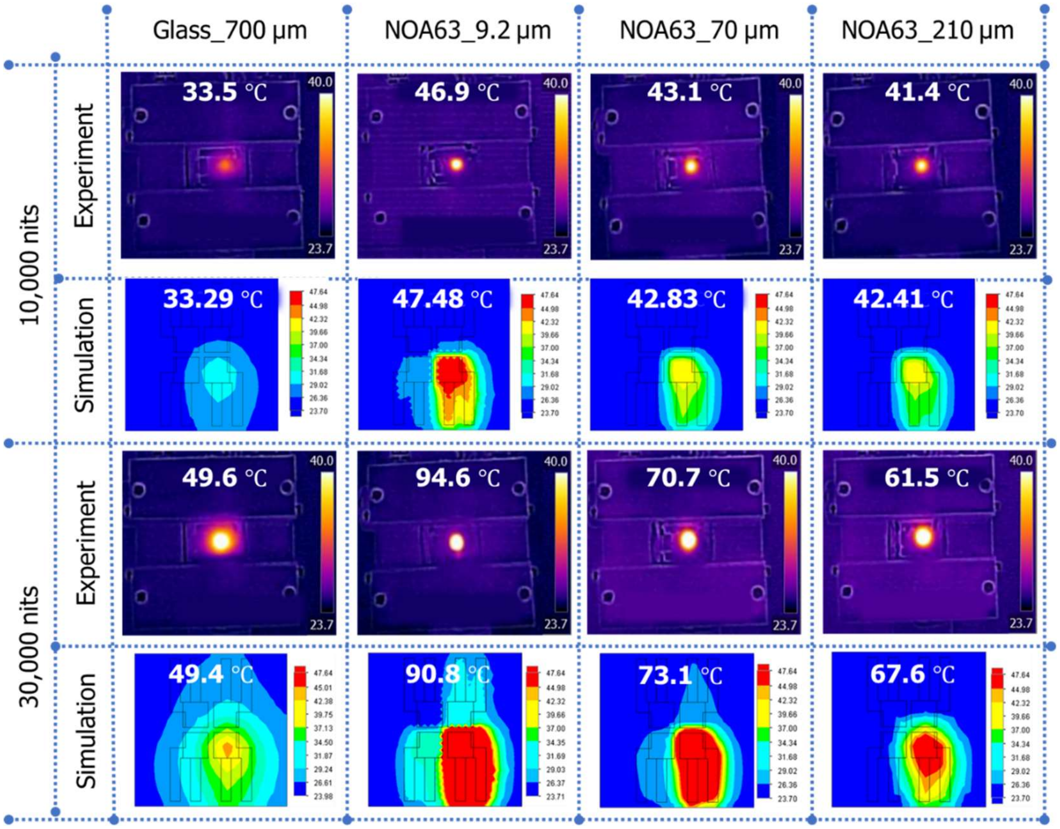

Figure 13. Thermal simulation and temperature observations from the real IR-camera images. The temperature observance of glass substrate (700 µm) and NOA63 substrate surfaces (9.2 µm, 70 µm, 210 µm) at 10,000 cd/m^2 and 30,000 cd/m^2. The heat spot size of the glass substrate is much wider, but not deeper than those of the NOA63 substrates, due to the difference between thermal conductivity and conductance as shown in Fig. 5b. The heat spread was wide, but not deeply distributed within the thick NOA63 and glass substrate. On the other hand, the heat spread was narrower but deep for thin NOA63 substrate. This is why the surface temperature of the thick glass substrate device (33.5°C) is lower than that of a the thin NOA63 substrate device (46.9°C) at 10,000 cd/m^2. While the surface temperature of the thick glass substrate device (49.6°C) is smaller than that of a thin NOA63 substrate device (94.6°C) at 30,000 cd/m^2 at high exciton density. As the values presented are for surface temperature, hence higher temperature in thin NOA63 elastomer surface are an indication that more heat is being exited from the devices as compared to glass substrate where the low surface temperature at the same exciton density indicates a smaller exit of heat.[28, 29] However, in the simulation results of the thin NOA63, hot spot size appeared wider because at the surface of NOA63, the heat is immediately distributed isotropically.

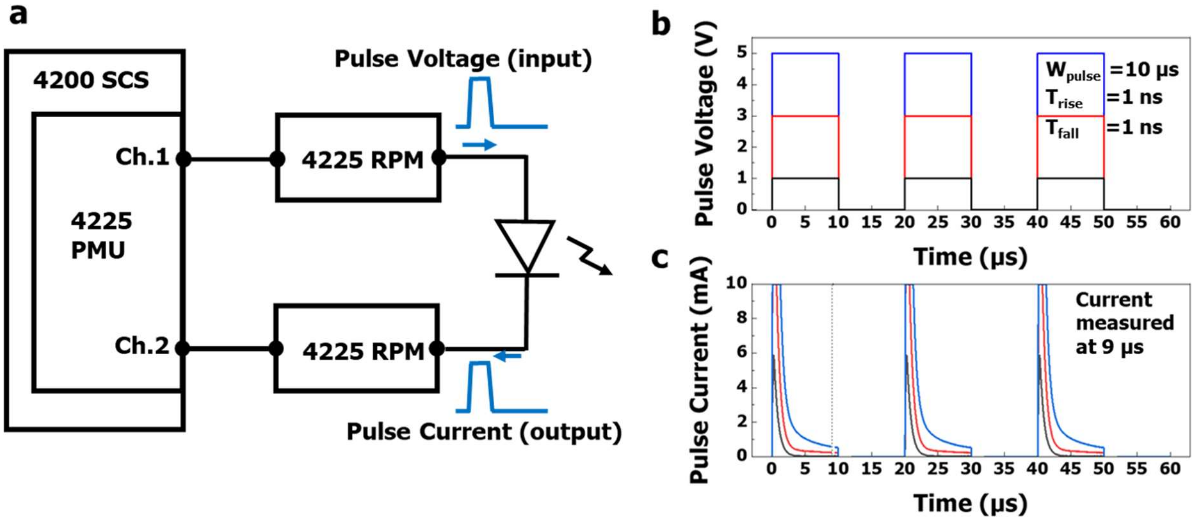

Figure 14. The pulsed measurement for heat dissipation properties of the GSOLEDs. a, The measurement schematic of pulse I-V using the Keithley 4200 Semiconductor Characterization System (SCS), 4225 Pulse Measurement Unit (PMU), and 4225 Remote Preamplifier (RPM). To manage the heat distribution of substrate, pulse I-V measurements were completed for each device. **b,** Transient pulse voltages at each input bias (1, 3, and 5 V). The voltage pulse condition was pulse width W_{pulse}=10 μs, rise time τ_{rise}=1 ns, fall time τ_{fall}=1 ns, and duty cycle=50 %. **c,** Output pulse current according to each pulse input. At the beginning of pulse, there was current overshooting. Pulse current was averaged using 3 measured current values at 9 μs from the start of each pulse, which shows suitable current saturation.

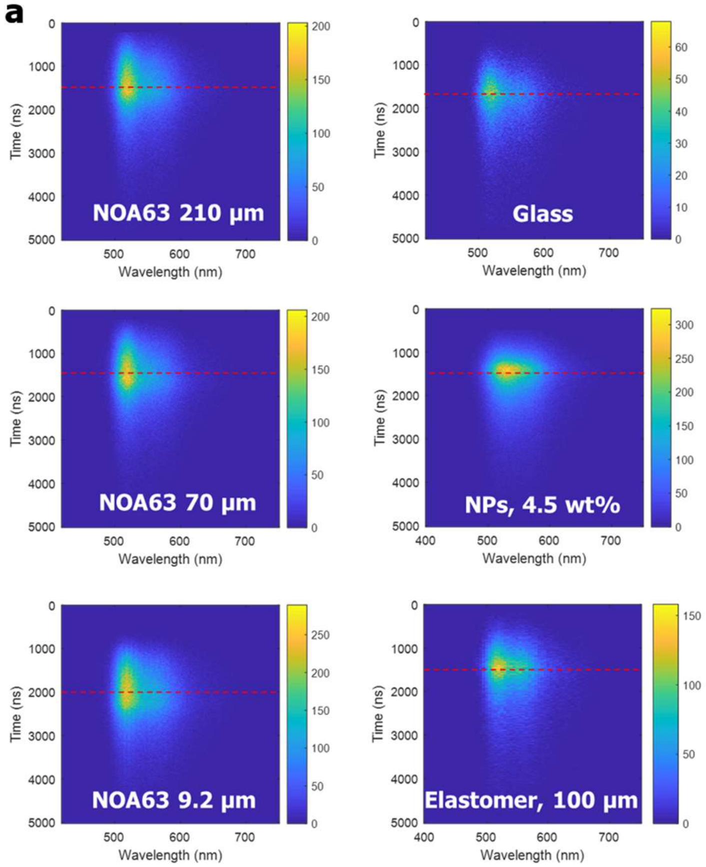

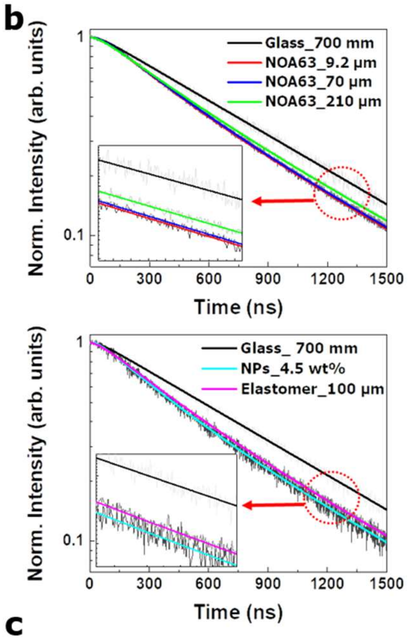

c

Sample	A_1 (%)	τ_1 (ns)	A_2 (%)	τ_2 (ns)
Glass			100	749
NOA63, 210 μm	46.6	438	53.4	884
NOA63, 70 μm	47.3	376	52.7	838
NOA63, 9.2 μm	39.2	325	60.8	788
NPs, 4.5 wt%	39.7	338	60.3	804
Elastomer, 100 μm	42.4	389	57.6	844

Figure 15. The time-resolved EL (TREL) measurement for verifying the effect of thicker film in heat dissipating. **a**, Time-resolved Electroluminescence (TREL) spectra in a 5 μs time window. **b**, Spectrally integrated TREL profiles that are truncated at the maximum EL intensity in the time-domain (dashed red lines around 1.5~2.0 μs in the raw data (a)), and the inset showed the magnified scale. **c,** Multi-exponential fitting results of the truncated TREL profiles. The TREL experimental results revealed that the EL lifetimes are sensitive to the thickness of substrate and the presence of additional dissipation pathways with NPs and thin elastomer. We believe that this observation is further evidence for supporting the heat dissipation mechanism for thin NOA63 devices where the faster exit of the heat reduced the TTA process. However, in the case of thick glass or thick NOA63 substrate, the heat was accumulated in the device and was mainly contributing to the TTA thus reducing the overall efficiency of the device. The efficient heat dissipation process may disturb any free-carrier (excitons) interactions such as TTA because exciton recombination occurring in lower energy states (trapping sites) become dominant in an environment with lower thermal energy.[30, 31, 32]

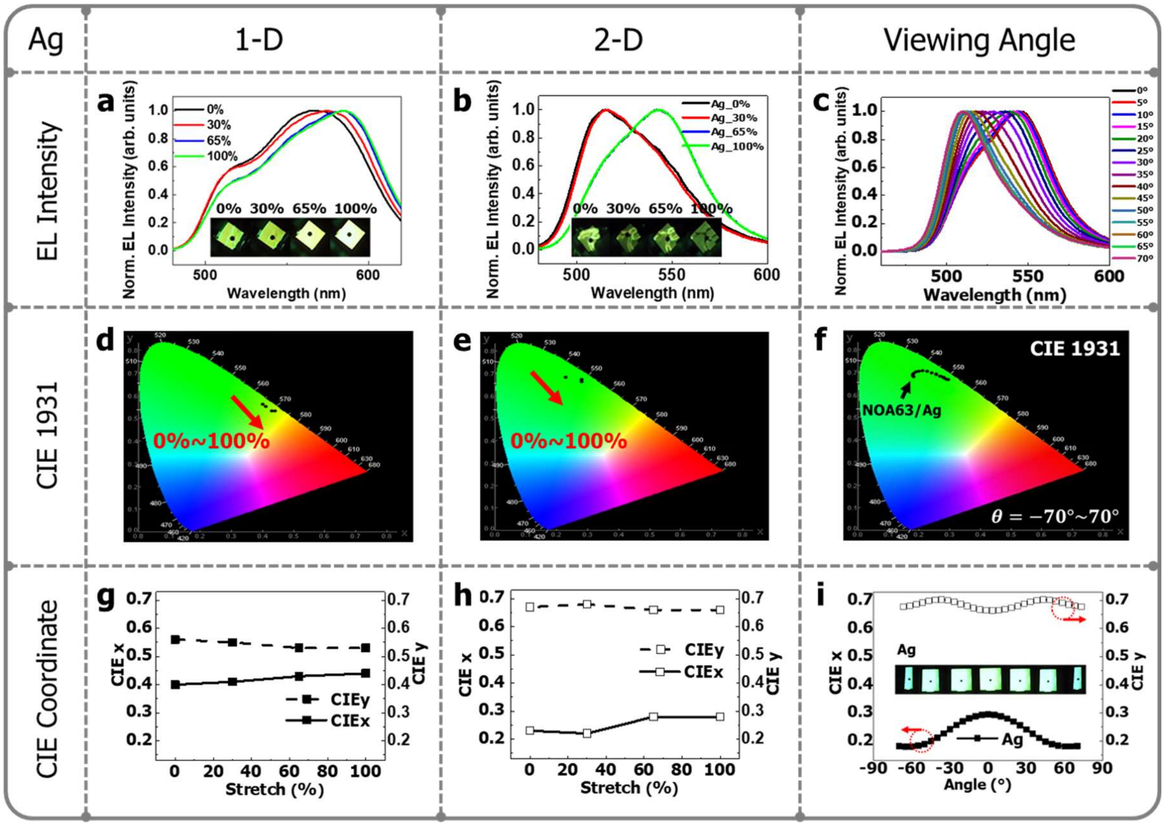

Figure 16. Chromic shifts in electroluminescence (EL) by the Ag electrode. **a-c,** 1D and 2D stretching at different percentages (0-100%) was applied to the fabricated GSOLED devices with Ag electrodes and the EL intensity was analysed. At 0% applied strain i.e. the buckled form of the device, the emission wavelength was found to be at ~560 nm which was shifted towards a slightly longer wavelength (yellow shift, ~570 nm) at 30% strain. When the devices were stretched to 65%, we speculate that the microcavity effect became dominant. This caused a further shift in the emission spectrum towards the longer wavelength (~590 nm) and this change in the emission colour could also be spotted from the camera images (shown as inset). **d-f,** Further, the CIE 1931 colour analysis was also found to be consistent with the obtained EL profile where with an increase in the applied strain an increase in the emission wavelength was observed. The viewing angle could also generate the microcavity and EL intensities are changeable as shown in Supplementary Figure 19c. **g-i,** The CIE colour system presented a relatively greener emission at 0% which became yellowish with the increase in strain percentage. With further increase in the applied strain to 100%, the emission wavelength was further yellow-shifted but with a minor difference, which indicates that the maximum effect of the microcavity was attained within the strain range of 65-100%.

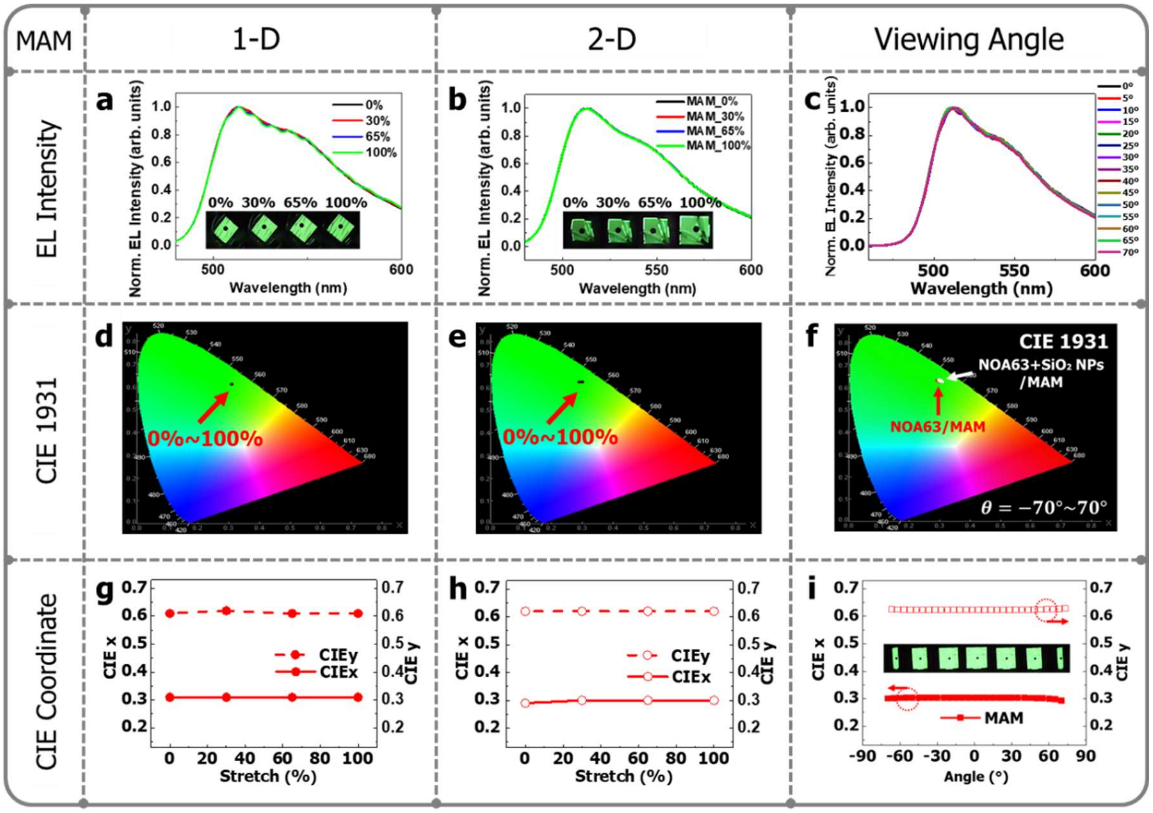

Figure 17. Electroluminescence (EL) intensity profiles for the MAM electrode devices. **a, b,** The EL intensity profile demonstrates the emission spectra for MAM based GSOLED devices. It could be observed that whether a 1D pre-strain was applied or 2D strain, the OLED devices presented a similar emission at ~510 nm. The EL spectra is in accordance with the camera images (shown as inset) of the pixels, where an identical luminance was maintained. The analysis suggests the absence of the microcavity effect in MAM devices and indicates that the MAM electrode was suitable for the fabrication of color-stable stretchable devices irrespective of the nature of the buckling mechanism i.e, 1D or 2D buckles. Furthermore, the color was stable, even at different strains, and is one of the most desirable properties in a geometrically stretchable OLEDs. This is because the previously presented top-emission buckled devices demonstrated an undesirable color-shift in the emissions with respect to the applied strain. **c,** The stable emission by the MAM GSOLED device was also analysed at variable angles (0-70°), where a negligible shift in the emission spectra was observed at any given angle. **d, e,** The CIE 1931 color coordinate analysis at various strain percentages (0-100%) showed a green emission for the MAM GSOLED devices which remained unchanged irrespective of the buckling mechanisms utilized i.e, 1D or 2D microcavity effect and the analysis is found in

accordance with the EL spectra. **f,** Further, the CIE 1931 at variable angles (-70 − 70°) also remained unchanged and was in accordance with the EL spectra. **g-i,** Respectively, the CIE 1931 for x and y-coordinates were without any changes in any specific axis and stable at any given variable angle. The analysis strongly indicated that the MAM-based OLED could provide an efficient solution for stretchable and wearable devices with high color stability.

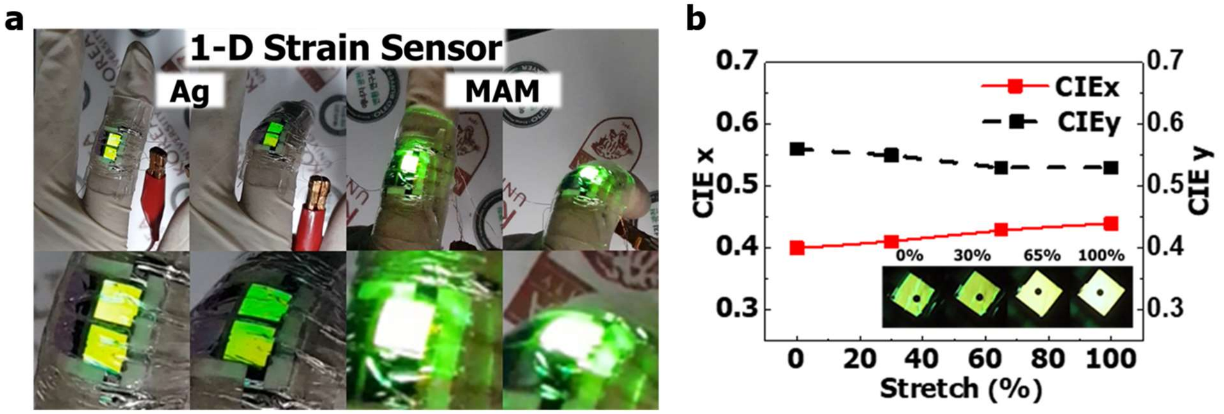

CIE x	0.4	0.41	0.43	0.44
Experiment value by strain %	0%	30%	65%	100%
Calculated value by strain %	0%	25%	75%	100%

CIE y	0.56	0.55	0.53	0.53
Experiment value by strain %	0%	30%	65%	100%
Calculated value by strain %	0%	33%	100%	100%

Strain %	0%	30%	65%	100%
Measured CIE x	0.4	0.41	0.43	0.44
Calculated CIE y	0.56	0.55	0.54	0.54

Strain %	0%	30%	65%	100%
Measured CIE y	0.56	0.55	0.53	0.53
Calculated CIE x	0.4	0.42	0.46	0.46

Figure 18. Devices with Ag electrode to demonstrate a shift in colour with respect to the applied strain motivating for an wearable strain sensor. a,b, The Ag device attached to the finger, where the straight position of the finger presented a yellow color that shifted to green upon bending, which was contrary to the green-yellow shift in the EL spectra of the devices due to the viewing angle of the observer. The x-CIE (*x*) and the y-CIE (*y*) are defined as,

$$x = m_x t + b_x \, , \tag{10}$$

$$y = m_y t + b_y \, , \tag{11}$$

where m_x and m_y are the slopes of *x* and *y* obtained by applying the fitting the values, *t* is the applied strain, and b_x is the intercept point of the slope on the y-axis. Similarly, the respective values for the y-CIE are presented in Equation (11).

$$t = \frac{1}{m_x} - b_x = \frac{1}{m_y} - b_y, \quad (12)$$

Here, the values of m_x and m_y were found to be 0.000418 and -0.000327, and b_x and b_y were 0.399 and 0.588, respectively. Hence, at any given strain value, the projection of the changes in x-CIE or y-CIE could be predicted, and vice versa. As from the experimental values, the x-CIE increases and y-CIE decreases with t, and the value of t at any given point in this relation remains constant, e.g, 25% for both x and y-axis, hence we could correlate the equations with t_c (constant strain). At any given value of strain (t_c), the x-axis is directly proportional and the y-axis is inversely proportional. Hence, the equation for each coordinate can be expressed as

$$x = -2y + 1.52 , \quad (13)$$

$$y = -\frac{1}{2}x + 0.76 . \quad (14)$$

Although the derived relationship was found to have some deviation in the results as compared to the experimental results, it could provide a rough estimation of the applied strain with respect to the measured x-CIE and y-CIE coordinates and could be helpful for understanding the dynamics of the Ag strain sensor.

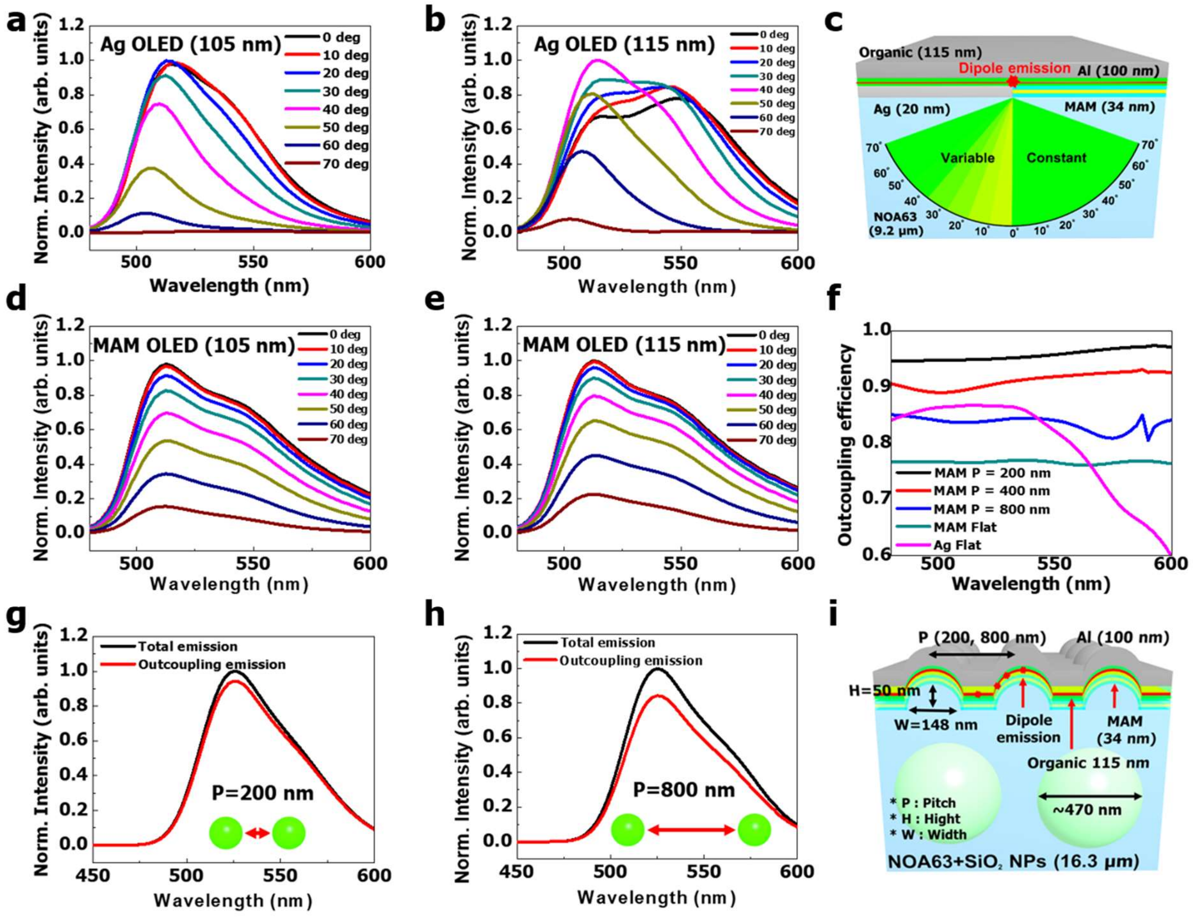

Figure 19. Optical and simulation analysis of the various mechanisms in the GSOLEDs. a-c, Optical simulation modelling is shown with the chromic shifts observed for the Ag electrode. As, the standard emission wavelength of the device is ~520 nm at normal angle (0°) using the 105 nm thick device, the observed emission for the Ag electrode was blue-shifted (towards the shorter wavelength region) with respect to an increase in measurement angle. This blue-shift in the emission follows the Fabry-Perot microcavity equation[33], given as:

$$\boldsymbol{\lambda_m} = \frac{4\pi nd\ cos\theta}{\phi_a+\phi_b-2\pi m}, \qquad (15)$$

where λ_m is the resonant wavelength, *n* is the refractive index, *d* is the physical thickness of the layer, θ is the angle in the space layer (measurement angle), ϕ_a *and* ϕ_b are the changes in the light phase when reflected from the interfaces a and b, respectively, and *m* is an integer (mode) number in the resonant condition. As discussed in the main manuscript, when θ increases, the value for $cos\theta$ decreases, which would consequently decrease the resulting λ_m. This implies that

the emission wavelength would present a blue-shift, which was observed for the Ag electrode OLEDs. Hence, the low transmittance and high reflectance of the Ag electrode, could account for the presence of microcavities in Ag-based devices. This effect was also observed due to the increased thickness (115 nm) of the OLED device with the Ag electrode. **d, e,** In contrast, the OLED devices with the MAM electrode, whether with 105 nm or 115 nm thickness, demonstrated negligible shifts in the emission wavelength regardless of the measurement angles. The phase changes (ϕ_a and ϕ_b) occurred when there was a significant reflection from the interfaces, which was not observed in the case of the MAM electrode. Hence, the negligible shift in wavelength indicates that the microcavity effect was insignificant. Therefore, the variable angle measurements for MAM OLEDs would not obey the Fabry-Perot microcavity equation. **f,** To analyze the effect of the high surface roughness (bumps) on the NOA63 substrate in an OLED device by the bumpy surface modification and agglomeration of NPs by the UV treatment, we assumed that the bumps were dispersed on a NOA63 surface. The bumps (50 nm height and 148 nm width) were present in a periodic manner with a given pitch, and then conformal OLED film structures were formed on a rough NOA63 surface. Finally, we calculated the out-coupling efficiency using the full-wave simulation (COMSOL Multiphysics, RF module) by taking the ratio of the extracted dipole emission light energy from the devices to the total emission light energy. Here, the simulation provided an interesting relationship between the pitch and the out-coupling efficiency. **g, h,** When the pitch was reduced, a significant increase in the out-coupling efficiency was observed, where a 200 nm pitch demonstrated the maximum value among all samples. This indicates that when the pitch became smaller, more light could be scattered from the bumpy structures of NOA63. We speculate that the light followed the forward Mie scattering[34] (for structures above 60 nm), which aided the extraction of more light out of the device instead of isotropic scattering (the Rayleigh scattering), in which case much light would have been dissipated. **i,** The schematic illustration of the bumpy structure with SiO_2 NPs including pitch, height and width.

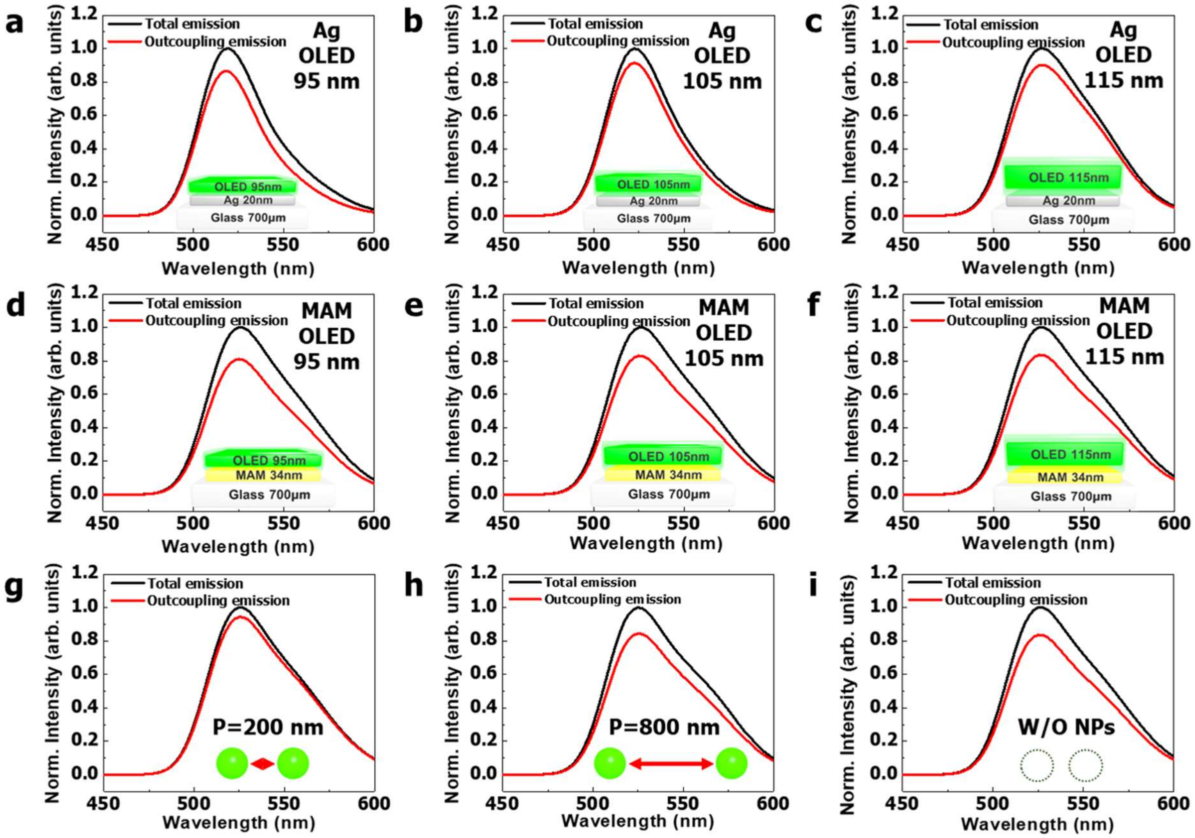

Figure 20. Calculated total and extracted light emission in GSOLEDs with MAM and Ag electrodes. **a-f,** The total and extracted light emission spectrum for GSOLED devices with an Ag electrode (a-c) and a MAM electrode (d-f). The thickness of the OLED active layer was 95, 105 and 115 nm. The out-coupling efficiency or intensity of the Ag device is enhanced due to the microcavity effect, as the OLED thickness is increased. The thickness of the MAM device can be considered almost identical from 95 nm to 105 and 115 nm due to absence of the microcavity effect. This indicates that the emission characteristics of the MAM electrode are preserved in the changes of the OLED structures, during stretching and bending. In contrast, the microcavity effect in the Ag electrode depends strongly on the emission characteristics, which is not desirable in the flexible display. **g-i,** The total and extracted light emission spectrum of the GSOLED devices when considering the bumpy surface (height 50 nm, width 148 nm) of the NOA63 were simulated. The thickness of the OLED active layer was 105 nm and the pitch of the periodic bump structures (P) were 200 (g) and 800 nm (h) (see Methods in the main manuscript). The out-coupling efficiency in MAM electrode can be enhanced by introducing periodic bumpy structures on the NOA63 surface with

the pitch of 200 and 800 nm. Compared to the OLED with the Ag electrode, the bump structures in the NOA63 with the pitch of 200 nm shows the enhanced extracted emission as well as the out-coupling efficiency. However, the extracted emission spectrum of the bump structure with a pitch of 800 nm in (h) is similar to the one without bump structures in (i). This indicates that a surface with a high surface roughness is effective at extracting the light from the OLED active layer by light scattering.[34] In addition, the total device performance with the MAM electrode was better than that of the Ag electrode due to better charge injection and balance with the MoO_3 thin dipole layer. Highly efficient light extraction from the OLED can be achieved with light scattering by the rough surface of NOA63 and better charge injection.

Table S1. Comparison among the GSOLEDs reported in articles[35, 36] and in this work.

Features	ACS Appl. Mater. Interfaces 2016, 8, 31166−31171 [35]	Organic Electronics 2017, 48, 314 [36]	This Work
Device Structure	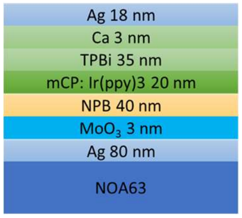	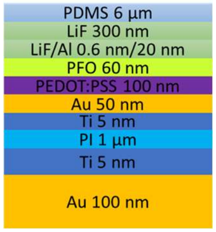	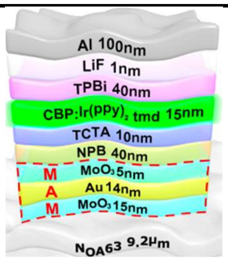
Elastomer	3M VHB (~ 1000 μm)	PDMS	Thin Elastomer 100 μm
Encapsulation (Waterproof)	X	X	SiN_x Passivated Film and Side NOA63 Encapsulation
Color Coordinate after Stretching	Shift	Shift	No Shift
Efficiency roll-off	O	O	X
Heat Dissipation	X	X	O (Thin elastomer or SiO_2 NPs)
Current Efficiency	71cd/A, @205cd/m^2	X	99.4cd/A, @20,000cd/m^2
Geometrical Stretchability	50%	3%	30% (Thin elastomer) 100% (3M elastomer)
Pre-Strain	Mechanically (100%)	Thermally (3%)	Mechanically (30%, 100%)
Mechanical Simulation	X	O	O
Optical Scattering	X	X	O

Table S2. Heat dissipating techniques used in OLEDs and their effect on device temperature, efficiency and lifetime. [29, 37, 38, 39, 40, 41]

Technique (Methods)	Temperature deviation compared to control device (ΔT, °C)	Efficiency improvement compared to control device (Δ cd/A, Δ)	Reference
Thin film encapsulation (Liquid getter)	7.7 °C @ 8 V	Not reported	[29, 37]
Heat sink (Cu layer)	8.5 °C and 11.5 °C @ 6 V and 9 V	Not reported	[38]
Heat sink (Heat transfer fluid)	Not measured	70% enhancement	[39]
Heat sink (Conducting substrate)	24 °C (for SUS) 43.7 °C (for silicon substrate)	3.4 % @ 8V (for SUS and silicon substrate)	[40]
Thin film encapsulation (Inorganic / organic / metal)	2.23 °C (with TFE) 4.51 °C (with MET-TFE)	Not reported	[41]
Heat dissipation (Thin substrate + SiO_2 NPs)	11.4 °C @ 10,000 nits	40% enhancement @ 10,000 nits	Current Work

Supplementary References

1. Guo YZ, Robertson J. Origin of the high work function and high conductivity of MoO3. *Appl Phys Lett* **105**, 222110 (2014).

2. Zhong JQ*, et al.* Ionization potential dependent air exposure effect on the MoO3/organic interface energy level alignment. *Organic Electronics* **13**, 2793-2800 (2012).

3. Kim M, Lim C, Jeong D, Nam H-S, Kim J, Lee J. Design of a MoOx/Au/MoOx transparent electrode for high-performance OLEDs. *Organic Electronics* **36**, 61-67 (2016).

4. Kim S, Lee J-L. Design of dielectric/metal/dielectric transparent electrodes for flexible electronics. *Journal of Photonics for Energy* **2**, 021215 (2012).

5. Cao W, Li J, Chen H, Xue J. Transparent electrodes for organic optoelectronic devices: a review. *Journal of Photonics for energy* **4**, 040990 (2014).

6. Davis C, McKenzie D, McPhedran R. Optical properties and microstructure of thin silver films. *Optics communications* **85**, 70-82 (1991).

7. Jalili S, Hajakbari F, Hojabri A. Effect of silver thickness on structural, optical and morphological properties of nanocrystalline Ag/NiO thin films. *Journal of Theoretical and Applied Physics* **12**, 15-22 (2018).

8. Hong K*, et al.* Optical properties of WO3/Ag/WO3 multilayer as transparent cathode in top-emitting organic light emitting diodes. *The Journal of Physical Chemistry C* **115**, 3453-3459 (2011).

9. Wrzesniewski E, Eom S-H, Hammond WT, Cao W, Xue J. Transparent oxide/metal/oxide trilayer electrode for use in top-emitting organic light-emitting diodes. *Journal of Photonics for Energy* **1**, 011023 (2011).

10. Kim S*, et al.* High-Performance Transparent Quantum Dot Light-Emitting Diode with Patchable Transparent Electrodes. *ACS applied materials & interfaces* **11**, 26333-26338 (2019).

11. Haacke G. New figure of merit for transparent conductors. *Journal of Applied Physics* **47**, 4086-4089 (1976).

12. Jeong S, Yoon H, Lee B, Lee S, Hong Y. Distortion-Free Stretchable Light-Emitting Diodes via Imperceptible Microwrinkles. *Advanced Materials Technologies* **5**, 2000231 (2020).

13. Kim SI, Lee KW, Sahu BB, Han JG. Flexible OLED fabrication with ITO thin film on polymer substrate. *Jpn J Appl Phys* **54**, (2015).

14. Liang J*, et al.* Silver nanowire percolation network soldered with graphene oxide at room temperature and its application for fully stretchable polymer light-emitting diodes. *ACS Nano* **8**, 1590-1600 (2014).

15. Carcia P, McLean R, Groner M, Dameron A, George S. Gas diffusion ultrabarriers on polymer substrates using Al 2 O 3 atomic layer deposition and SiN plasma-enhanced chemical vapor deposition. *Journal of Applied Physics* **106**, 023533 (2009).

16. Lee L*, et al.* Ultra gas-proof polymer hybrid thin layer. *Nano letters* **18**, 5461-5466 (2018).

17. Park J, Yoon HR, Khan MA, Cho S, Sung MM. Selective Infiltration in Polymer Hybrid Thin Films as a Gas-Encapsulation Layer for Stretchable Electronics. *ACS Applied Materials & Interfaces* **12**, 8817-8825 (2020).

18. Park M*, et al.* Mechanically recoverable and highly efficient perovskite solar cells: investigation of intrinsic flexibility of organic–inorganic perovskite. *Advanced Energy Materials* **5**, 1501406 (2015).

19. Li YF*, et al.* Stretchable Organometal-Halide-Perovskite Quantum-Dot Light-Emitting Diodes. *Adv Mater* **31**, e1807516 (2019).

20. Yin D*, et al.* Roller-Assisted Adhesion Imprinting for High-Throughput Manufacturing of Wearable and Stretchable Organic Light-Emitting Devices. *Advanced Optical Materials* **8**, (2019).

21. Jeong S, Yoon H, Lee B, Lee S, Hong Y. Distortion-Free Stretchable Light-Emitting Diodes via Imperceptible Microwrinkles. *Advanced Materials Technologies* **5**, (2020).

22. Oh M-C, Park J-H, Jeon HJ, Go JS. Hollow-core polymeric nanoparticles for the enhancement of OLED outcoupling efficiency. *Displays* **37**, 72-78 (2015).

23. Arzenšek D, Podgornik R, Kuzman D. Dynamic light scattering and application to proteins in solutions. In: *Seminar; University of Ljubljana: Ljubljana, Slovenia*) (2010).

24. Efimenko K, Wallace WE, Genzer J. Surface modification of Sylgard-184 poly (dimethyl siloxane) networks by ultraviolet and ultraviolet/ozone treatment. *Journal of colloid and interface science* **254**, 306-315 (2002).

25. Hillborg H, Tomczak N, Olàh A, Schönherr H, Vancso GJ. Nanoscale hydrophobic recovery: A chemical force microscopy study of UV/ozone-treated cross-linked poly (dimethylsiloxane). *Langmuir* **20**, 785-794 (2004).

26. Wong Y, Pellegrino S. Computation of wrinkle amplitudes in thin membrane. In: *43rd AIAA/ASME/ASCE/AHS/ASC Structures, Structural Dynamics, and Materials Conference*) (2002).

27. Park JH*, et al.* Flash-Induced Stretchable Cu Conductor via Multiscale-Interfacial Couplings. *Advanced Science* **5**, 1801146 (2018).

28. Park J, Ham H, Park C. Heat transfer property of thin-film encapsulation for OLEDs. *Organic Electronics* **12**, 227-233 (2011).

29. Tyagi P, Srivastava R, Giri LI, Tuli S, Lee C. Degradation of organic light emitting diode: Heat related issues and solutions. *Synthetic Metals* **216**, 40-50 (2016).

30. Lee KH, Lee JY. Phosphor sensitized thermally activated delayed fluorescence organic light-emitting diodes with ideal deep blue device performances. *Journal of Materials Chemistry C* **7**, 8562-8568 (2019).

31. Ràfols-Ribé J*, et al.* High-performance organic light-emitting diodes comprising ultrastable glass layers. *Science advances* **4**, eaar8332 (2018).

32. Yoshida K, Nakanotani H, Adachi C. Effect of Joule heating on transient current and electroluminescence in pin organic light-emitting diodes under pulsed voltage operation. *Organic Electronics* **31**, 287-294 (2016).

33. Jung BY, Kim NY, Lee C, Hwangbo CK. Control of resonant wavelength from organic light-emitting materials by use of a Fabry-Perot microcavity structure. *Applied optics* **41**, 3312-3318 (2002).

34. Zhou J*, et al.* Roughening the white OLED substrate's surface through sandblasting to improve the external quantum efficiency. *Organic Electronics* **12**, 648-653 (2011).

35. Yin D, Feng J, Jiang N-R, Ma R, Liu Y-F, Sun H-B. Two-dimensional stretchable organic light-emitting devices with high efficiency. *ACS applied materials & interfaces* **8**, 31166-31171 (2016).

36. Hafeez H*, et al.* Multiaxial wavy top-emission organic light-emitting diodes on thermally prestrained elastomeric substrates. *Organic Electronics* **48**, 314-322 (2017).

37. Ham H, Park J, Kim Y. Thermal and barrier properties of liquid getter-filled encapsulations for OLEDs. *Organic Electronics* **12**, 2174-2179 (2011).

38. Choi SH, Lee TI, Baik HK, Roh HH, Kwon O, Suh Dh. The effect of electrode heat sink in organic-electronic devices. *Appl Phys Lett* **93**, (2008).

39. Zakhidov AA, Reineke S, Lüssem B, Leo K. Hydrofluoroethers as heat-transfer fluids for OLEDs: Operational range, stability, and efficiency improvement. *Organic Electronics* **13**, 356-360 (2012).

40. Chung S, Lee J-H, Jeong J, Kim J-J, Hong Y. Substrate thermal conductivity effect on heat dissipation and lifetime improvement of organic light-emitting diodes. *Appl Phys Lett* **94**, (2009).

41. Zhang W*, et al.* Heat Dissipation Properties of Thin-Film Encapsulation by Insertion of a Metal Thin Film for Organic Light-Emitting Diodes. *physica status solidi (a)* **215**, (2018).
