# Supplementary material for: Highly efficient, heat dissipating, stretchable organic light-emitting diodes based on a MoO3/Au/MoO3 electrode with encapsulation
Source: Nat Commun. 2021 May 17;12:2864. doi: 10.1038/s41467-021-23203-y (PMC8128878; doi:10.1038/s41467-021-23203-y)
Supplement: Supplementary file 3 — Description of Additional Supplementary Files [file 41467_2021_23203_MOESM3_ESM.pdf]

Description of Additional Supplementary Files

File Name: Supplementary Movie 1
Description: two-dimensional stretching mode

File Name: Supplementary Movie 2
Description: peel-off process

File Name: Supplementary Movie 3
Description: twisting mode-1

File Name: Supplementary Movie 4
Description: twisting mode-2

File Name: Supplementary Movie 5
Description: convex mode

File Name: Supplementary Movie 6
Description: (water immersion mode

File Name: Supplementary Movie 7
Description: wearable strain sensor based on Ag

File Name: Supplementary Movie 8
Description: wearable strain sensor based on MAM
